# Efficacy and safety of GLP-1RAs, SGLT2is, and nsMRAs for cardiovascular and renal outcomes in T2DM with CKD: a systematic review and network meta-analysis

**DOI:** 10.3389/fendo.2026.1937754

**Published:** 2026-09-08

**Authors:** Hongli Chen, Xuejie Wu, Mengyi Yuan, Feihong Ren, Tongtong Liu, Liping Yang, Xuedong An, Yongli Zhan

**Affiliations:** 1 Guang’anmen Hospital, China Academy of Chinese Medical Sciences, Beijing, China; 2 Graduate School, Beijing University of Chinese Medicine, Beijing, China

**Keywords:** cardiovascular outcomes, chronic kidney disease, GLP-1 receptor agonists, network meta-analysis, nsMRA, renal outcomes, SGLT2 inhibitors, type 2 diabetes mellitus

## Abstract

**Objective:**

To compare cardiovascular and renal benefits and safety of GLP-1RAs, SGLT2is, and nsMRAs in T2D-CKD using network meta-analysis.

**Methods:**

RCTs published through 7 August 2026 were systematically identified. A frequentist random-effects network meta-analysis was performed. Evidence certainty was assessed with CINeMA.

**Results:**

A total of 21 RCTs involving 51,899 patients were included. Compared with placebo, nsMRAs were associated with lower risks of the major kidney composite outcome and hospitalization for heart failure (HHF), whereas SGLT2is were associated with lower risks of major adverse cardiovascular events (MACE), the major kidney composite outcome, and HHF. GLP-1RAs were associated with lower risks of MACE, the major kidney composite outcome, HHF, all-cause mortality, and cardiovascular death. Between-class indirect comparisons suggested a lower estimated risk of the major kidney composite outcome with SGLT2is than with GLP-1RAs (RR 0.77, 95% CI 0.64–0.91) or nsMRAs (nsMRAs versus SGLT2is: RR 1.28, 95% CI 1.10–1.50). The estimated risk of HHF was also lower with SGLT2is than with nsMRAs (nsMRAs versus SGLT2is: RR 1.28, 95% CI 1.01–1.63). For cardiovascular death, the indirect estimate suggested a lower risk with GLP-1RAs than with SGLT2is (SGLT2is versus GLP-1RAs: RR 1.28, 95% CI 1.03–1.58), whereas the comparison between nsMRAs and GLP-1RAs did not reach statistical significance (RR 1.24, 95% CI 0.99–1.56). No statistically significant between-class differences were identified for MACE or all-cause mortality. All between-class comparisons were based exclusively on indirect evidence. Safety analyses showed higher risks of hyperkalemia with nsMRAs and genital infections with SGLT2is than with placebo. Sensitivity analyses broadly supported the main findings. Confidence in the evidence was mostly low to moderate, primarily because of indirectness.

**Conclusion:**

In patients with T2D-CKD, all three drug classes reduced several cardiorenal outcomes versus placebo. Between-class indirect comparisons suggested more favorable relative-effect estimates for SGLT2is on major kidney outcomes and HHF in selected comparisons, and for GLP-1RAs on cardiovascular death. No clear between-class difference was observed for MACE. Because the network lacked head-to-head comparisons between active drug classes, these findings and treatment rankings should be interpreted cautiously. Direct comparative and combination-therapy trials are needed to guide individualized treatment selection.

**Systematic Review Registration:**

https://www.crd.york.ac.uk/PROSPERO/view/CRD420251150683, identifier CRD420251150683.

## Introduction

Chronic kidney disease (CKD) is one of the most common complications in patients with type 2 diabetes mellitus (T2DM). Globally, the epidemiological burden of type 2 diabetes with chronic kidney disease (T2D-CKD) is increasingly severe. A meta-analysis of 1,711,926 patients reported that the prevalence of CKD among patients with T2DM is as high as 27% (1). Moreover, T2D-CKD is associated with an increased risk of mortality. Previous cohort studies have demonstrated that the 10-year all-cause mortality rate in patients with T2D-CKD is approximately three times higher than in diabetes alone (2). The natural course of T2D-CKD involves glomerular hyperfiltration, persistent proteinuria, and progressive decline in glomerular filtration rate, ultimately leading to end-stage renal disease, driven by diabetes-related metabolic disturbances that cause glomerular hypertrophy or sclerosis, as well as tubular interstitial inflammation and fibrosis (3).

In previous clinical practice, renin–angiotensin system inhibitors (RASi) have long served as the cornerstone therapy for T2D-CKD. However, substantial residual cardiorenal risk persists in patients with T2D and CKD despite treatment with RASi (4). In recent years, sodium–glucose cotransporter 2 inhibitors (SGLT2is), glucagon-like peptide-1 receptor agonists (GLP-1RAs), and nonsteroidal mineralocorticoid receptor antagonists (nsMRAs) have demonstrated additional cardiorenal protective effects in randomized controlled trials. These agents have been progressively incorporated into clinical guidelines or consensus recommendations and have become important therapeutic options in the comprehensive management of T2DM with CKD (5).

Mechanistically, the three drug classes target complementary processes that drive CKD progression in T2D. SGLT2is inhibit proximal tubular sodium–glucose cotransport, increase sodium delivery to the macula densa, and restore tubuloglomerular feedback. These effects reduce intraglomerular pressure and glomerular hyperfiltration, while glycosuria, natriuresis, and reduced tubular workload mitigate metabolic and hypoxic stress, thereby reducing albuminuria and slowing kidney function decline (6). GLP-1RAs reduce metabolic stress by improving glycemic control and reducing body weight and blood pressure. Their natriuretic, anti-inflammatory, antioxidant, and vascular effects may further attenuate glomerular and tubulointerstitial injury (7). By contrast, nsMRAs do not primarily lower blood glucose but block pathological mineralocorticoid receptor activation, thereby limiting sodium retention, renal inflammation, fibrosis, and maladaptive tissue remodeling. When added to standard renin–angiotensin system inhibition, these effects can reduce albuminuria and slow CKD progression (8). These distinct yet complementary mechanisms may underlie different patterns of cardiorenal benefit.

However, head-to-head RCTs comparing SGLT2is, GLP-1RAs, and nsMRAs are lacking. Network meta-analysis can estimate their relative effects indirectly by linking trials through a common comparator. A previous network meta-analysis found that SGLT2is and nsMRAs significantly reduced composite renal outcomes compared with placebo, whereas GLP-1RAs did not show a statistically significant reduction (RR 0.90, 95% CI 0.80–1.02) (9). The subsequent FLOW trial, the first dedicated kidney outcomes trial of a GLP-1RA, showed that semaglutide reduced major renal events in patients with T2D and CKD (10). Whether incorporation of this new evidence changes previous comparative estimates remains unclear. Metabolic parameters, including HbA1c, body weight, and blood pressure, are relevant to long-term cardiorenal risk and individualized treatment decisions but have rarely been integrated into comparative analyses.

Therefore, we conducted a systematic review and network meta-analysis including the latest FLOW trial to compare cardiovascular, renal, and safety outcomes of the three drug classes in T2DM with CKD, along with an exploratory network analysis of metabolic parameters to inform individualized treatment.

## Methods

### Protocol and registration

This systematic review and network meta-analysis was performed and reported in accordance with the PRISMA 2020 statement, PRISMA-NMA extension, and Cochrane Handbook for Systematic Reviews of Interventions (11, 12). The study was prospectively registered in PROSPERO (CRD420251150683; 19 September 2025).

### Search strategy and information sources

The search and study selection followed the PICOS framework using subject headings and free-text keywords. A comprehensive search was performed in PubMed, Embase, the Cochrane Library, and Web of Science from database inception to 7 August 2026. The detailed strategy is provided in **Supplementary Material S1**. Two reviewers (C and W) independently screened records in duplicate with blinding; disagreements were resolved by a third reviewer (A). Reference lists of included studies were manually checked to avoid omissions.

### Eligibility criteria

Studies were included if they met the following criteria: Published in peer-reviewed journals; Population: adults (age ≥18 years) diagnosed with T2DM and CKD, defined according to KDIGO 2024 as: (a) urinary albumin-to-creatinine ratio (UACR) ≥30 mg/g; and/or (b) estimated glomerular filtration rate (eGFR) <60 mL/min/1.73 m² for ≥3 months; or (c) other evidence of kidney damage (e.g., abnormal urinalysis, imaging findings, or renal biopsy consistent with diabetic kidney disease); Study design: randomized controlled trials (RCTs), with: Intervention group: treatment with at least one of the following target agents—nsMRAs, SGLT2is, or GLP-1RAs; Control group: placebo or another target drug listed above; Follow-up duration of at least 24 weeks; Reporting at least one extractable prespecified outcome.

### Exclusion criteria

Duplicate publications; non-RCTs; non-English articles; animal or observational studies; reviews; case reports; retrospective analyses; incomplete original data; conference abstracts; no accessible full text. Also excluded: patients on dialysis or post-kidney transplantation; major non-diabetic kidney disease; type 1 diabetes; intervention groups adding other glucose-lowering or renoprotective agents beyond the three drug classes (effects inseparable); control groups receiving active agents (not placebo/standard care) that substantially affect renal outcomes; and missing key baseline/follow-up data unobtainable from authors.

### Data extraction and processing

Two reviewers (C and W) independently extracted data from the included studies using a predefined and pilot-tested standardized data extraction form. Based on the PICOS framework, the extracted information included: study characteristics (trial name, publication year, registration number, country), population and baseline characteristics (sample size, age, sex, baseline eGFR, UACR, HbA1c, BMI, duration of diabetes, and renal function criteria used in the original study), interventions and comparators (drug class, specific agent, dosage, comparator), follow-up duration, proportion of RASi use, outcome definitions, and risk of bias assessment.

Outcome data were extracted according to the prespecified endpoint definitions. For dichotomous outcomes, the numbers of events and participants in each group were extracted. For continuous outcomes, the sample size, mean, and standard deviation (SD) were extracted, with change-from-baseline data preferred when available. When an SD was not reported, it was derived from the available summary statistics. For a group mean or mean change, the SD was calculated from the standard error (SE) as


$$SD=SE\sqrt{n},$$

or from the corresponding two-sided 95% confidence interval (CI) as


$$SD=\frac{(U-L)\sqrt{n}}{2t_{0.975,n-1}},$$

Where n denotes the group sample size, *U* and *L* denote the upper and lower confidence limits, respectively, and $t_{0.975,n-1}\;$is the corresponding critical value of the t-distribution. For large samples, $2t_{0.975,n-1}$was approximated by 3.92. When a 95% CI was reported for a between-group mean difference, the SE of the mean difference was first calculated as


$$SE_{\text{MD}}=\frac{U-L}{2t_{0.975,df}},$$

Where *df* denotes the corresponding degrees of freedom. Assuming equal within-group variances, the common within-group SD was then estimated as


$$SD_{\text{pooled}}=\frac{SE_{\text{MD}}}{\sqrt{1/n_{1}+1/n_{2}}}.$$

Data at 24 weeks were preferentially extracted. When 24-week data were unavailable, data from the closest reported time point were used; for trials reporting only longer-term metabolic assessments, the available long-term assessment was extracted. For trials containing multiple eligible dose arms of the same drug, such as canagliflozin 100 mg and 300 mg in Yale (13), these arms were combined into a single treatment node, and the shared comparator was included only once. For dichotomous outcomes, event counts and sample sizes were summed across the eligible dose arms. For continuous outcomes, the combined sample size and mean were calculated as


$$N=\sum_{i=1}^{k}n_{i},\;\bar{X}=\frac{\sum_{i=1}^{k}n_{i}{\bar{X}}_{i}}{N},$$

and the combined SD was calculated as


$$SD_{\text{combined}}=\sqrt{\frac{\sum_{i=1}^{k}(n_{i}-1)SD_{i}^{2}+\sum_{i=1}^{k}n_{i}{\left({\bar{X}}_{i}-\bar{X}\right)}^{2}}{N-1}}.$$

For studies presenting results only in graphical form (n = 1), data were extracted using graphical digitization software (WebPlotDigitizer, version 4.6, https://automeris.io/WebPlotDigitizer), and the average of the two reviewers’ (C and W) extracted values was used. Discrepancies between reviewers were resolved through discussion; if consensus could not be reached, a third reviewer (A) adjudicated.

For large cardiovascular outcome trials not enrolling T2D-CKD patients, we extracted data from published subgroup analyses focusing on the CKD population [e.g., liraglutide data from the LEADER trial were taken from the subgroup analysis by Mann et al. (14) rather than the main trial report (15)] to ensure alignment with our target population.

### Transitivity assessment

The transitivity assumption was evaluated by descriptively comparing the distribution of potential effect modifiers across treatment comparisons.

### Data extraction and quality assessment

Two reviewers (C and W) independently extracted data and assessed the risk of bias of eligible studies using the Cochrane Risk of Bias tool (RoB 2.0) (16). Any disagreements in data extraction or quality assessment were resolved by a third reviewer (A).

To evaluate the certainty of evidence for the five prespecified clinical hard endpoints in the network meta-analysis, the Confidence in Network Meta-Analysis (CINeMA) framework was applied. Metabolic outcomes were considered exploratory and were not included in the CINeMA assessment. Following CINeMA guidelines, each comparison was assessed across six domains: within-study bias, reporting bias, indirectness, imprecision, heterogeneity, and incoherence (17, 18). The specific criteria for the six dimensions can be found in the **Supplementary Materials**.

### Outcomes and outcome definitions

This study evaluated five main outcomes: major adverse cardiovascular events (MACE), the major kidney composite outcome, hospitalization for heart failure (HHF), cardiovascular death (CVD), and all-cause mortality (ACD). MACE was defined according to the prespecified definition in each trial and generally comprised cardiovascular death, nonfatal myocardial infarction, and nonfatal stroke. The major kidney composite outcome was also extracted according to each trial’s prespecified definition and generally included a sustained ≥40% or ≥50% decline in eGFR or doubling of serum creatinine, kidney failure or end-stage kidney disease, kidney replacement therapy, or renal death. Event counts were not converted across different definitions. Exploratory meta-analyses of HbA1c, body weight, and systolic blood pressure were additionally performed to complement the hard-endpoint evidence.

For the major kidney composite outcome, we retained each trial’s prespecified definition and recorded its individual components during data extraction. Event counts were extracted as reported and were not recalculated or converted across different endpoint definitions.

### Statistical analysis

Network meta-analysis was performed in Stata 18.0 using a frequentist framework and the official network commands for data preparation, model fitting, and plot generation (network map). Node size reflects cumulative sample size; edge thickness indicates the number of direct comparisons. For dichotomous outcomes, risk ratios (RRs) with 95% CI were used. Given expected clinical and methodological heterogeneity across large outcome trials, a random-effects consistency model was applied, with between-study variance (τ²) estimated by restricted maximum likelihood.

Between-study heterogeneity was assessed using the Q test, τ², and approximate I², reported alongside results. Since all studies shared placebo as the common comparator with no head-to-head trials among active drug classes, no closed loops formed. Thus, all between-class comparisons relied on indirect evidence; inconsistency testing was not applicable, and network synthesis was performed under the consistency assumption.

SUCRA values were calculated under the random-effects model to provide reference information on relative treatment rankings and were interpreted alongside the effect estimates and their 95% CIs. League tables were generated to present all pairwise network estimates, with placebo as the reference treatment (RR = 1). To explore within-class differences and supplement class-level conclusions, treatment nodes were further disaggregated by individual agents, and drug-level network meta-analyses were conducted.

Exploratory continuous outcomes, including body weight, HbA1c, and systolic blood pressure, were expressed as mean differences (MDs) with 95% CIs. When the available evidence was insufficient to construct a connected network encompassing all three drug classes, conventional random-effects meta-analyses of active treatment versus placebo were performed, with analyses stratified by drug class. Subgroup forest plots were generated, and differences between drug-class subgroups were tested when estimable.

As the network contained no direct head-to-head comparisons among active treatments and no closed loops, statistical assessment of inconsistency was not feasible.

### Safety analysis

For safety outcomes, if evidence was limited to single direct comparisons or insufficient node coverage, traditional random-effects pairwise meta-analysis or descriptive summaries were used.

### Sensitivity analyses

To assess the robustness of the main findings and whether differences between CKD-specific trials and CKD subgroup data derived from CVOTs materially influenced the comparative estimates, *post hoc* sensitivity analyses were conducted for MACE and the major kidney composite outcome by repeating the network meta-analysis separately within each data source. For the major kidney composite outcome, the influence of variations in kidney outcome definitions was also evaluated.

## Results

### Study selection and included studies

**Figure 1:**
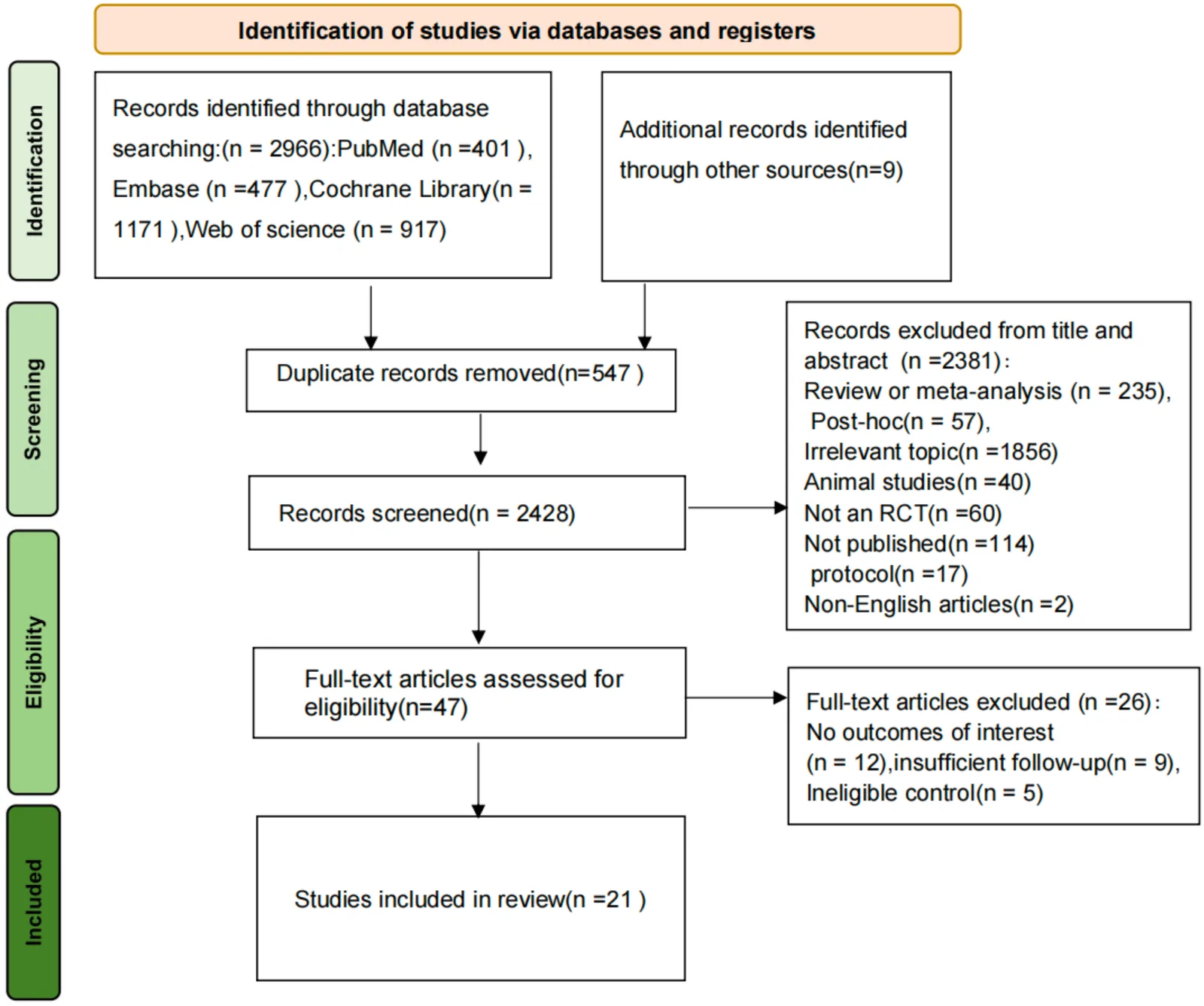
PRISMA flowchart. For some cardiovascular outcome trials (CVOTs), CKD-related outcome data were derived from prespecified subgroup or secondary analyses. These publications were cited solely as data sources and were not counted as separate trials.

The process of literature search and study selection is shown in **Figure 1**. A total of 21 RCTs, involving 51,899 patients with T2D-CKD, were ultimately included in the quantitative synthesis. A total of 2,966 records were identified through systematic database searches, including PubMed (n = 401), the Cochrane Library (n = 1,171), Web of Science (n = 917), and Embase (n = 477). An additional nine records were identified through screening the reference lists of relevant meta-analyses. After removing 547 duplicate records, 2,428 records remained for screening. Following title and abstract screening, 2,381 records were excluded for the following reasons: reviews or meta-analyses (n = 235), irrelevant topics (n = 1,856), *post hoc* analyses (n = 57), animal studies (n = 40), non-RCTs (n = 60), unpublished studies (n = 114), study protocols (n = 17), and non-English articles (n = 2). A total of 47 full-text articles were assessed for eligibility. After full-text evaluation, 12 studies were excluded because they did not report outcomes of interest, five because they used ineligible interventions or comparators, and nine because of insufficient follow-up duration. The updated search did not identify any additional published RCTs that met all eligibility criteria. Ultimately, 21 RCTs were included in this network meta-analysis (10, 13, 15–42). Among the 21 included studies, two trials compared finerenone (19, 20) with placebo; six trials evaluated GLP-1 receptor agonists, including liraglutide (15), injectable semaglutide (10), exenatide (21, 22), dulaglutide (23, 24), and oral semaglutide (25), versus placebo. Thirteen trials evaluated SGLT2 inhibitors, including empagliflozin (26–28), dapagliflozin (29–34), canagliflozin (13, 35–38), bexagliflozin (39), ertugliflozin (40, 41), and sotagliflozin (42), compared with placebo.

### Study characteristics and network structure

A total of 21 RCTs were included, covering three classes of targeted therapies: SGLT2is, GLP-1RAs, and nsMRAs. All controls received placebo. Among 51,899 T2D-CKD patients, 26,394 were in intervention groups and 25,505 in control groups. All trials were multicenter and multinational, with follow-up ranging from 24 weeks to 5 years (**Table 1**).

**Table 1:** Baseline characteristics of included studies in patients with T2DM and CKD.

Trial,year	ClinicalTrials.gov number	Country	Number of patients			Mean age		Male (%)		Baseline eGFR (ml/min/1.73 m^2^)		HbA1c (%)		Baseline BMI (kg/m²)		Duration of diabetes (y)	
			Total	I	C	I	C	I	C	I	C	I	C	I	C	I	C
nsMRA vs placebo																	
FIDELIO-DKD,2020	NCT02540993	Multinational	5,674	2,833	2,841	65.4 ± 8.9	65.7 ± 9.2	68.9	71.5	44.4 ± 12.5	44.3 ± 12.6	7.7 ± 1.3	7.7 ± 1.4	31.1 ± 6.0	31.1 ± 6.0	16.6 ± 8.8	16.6 ± 8.8
FIGARO-DKD,2021	NCT02545049	Multinational	7,352	3,686	3,666	64.1 ± 9.7	64.1 ± 10.0	68.6	70.3	67.6 ± 21.7	68.0 ± 21.7	7.7 ± 1.4	7.7 ± 1.4	31.5 ± 6.0	31.4 ± 5.9	14.5 ± 8.6	14.4 ± 8.4
GLP-1-RA vs placebo																	
LEADER,2016	NCT01179048	Multinational	2,158	1,116	1,042	67.3 ± 7.5	67.3 ± 7.5	61.9	60.6	45.5 ± 10.9	45.8 ± 10.8	8.7 ± 1.6	8.6 ± 1.5	32.6 ± 6.4	32.7 ± 6.6	15.4 ± 8.7	14.9 ± 8.5
SUSTAIN-6,2016	NCT01720446	Multinational	939	469	470	N/A	N/A	N/A	N/A	N/A	N/A	N/A	N/A	N/A	N/A	N/A	N/A
EXSCEL,2017	NCT01144338	Multinational	3,177	1,557	1,620	N/A	N/A	N/A	N/A	N/A	N/A	N/A	N/A	N/A	N/A	N/A	N/A
REWIND,2019	NCT01394952	Multinational	2,199	1,081	1,118	N/A	N/A	N/A	N/A	N/A	N/A	N/A	N/A	N/A	N/A	N/A	N/A
PIONEER 6,2019	NCT02692716	Multinational	856	434	422	N/A	N/A	N/A	N/A	N/A	N/A	N/A	N/A	N/A	N/A	N/A	N/A
FLOW,2024	NCT03819153	Multinational	3,533	1,767	1,766	66.6 ± 9.0	66.7 ± 9.0	70.6	68.9	46.9 ± 15.6	47.1 ± 14.7	7.8 ± 1.3	7.8 ± 1.3	31.9 ± 6.1	32.0 ± 6.5	N/A	N/A
SGLT-2 inhibitors vs. placebo																	
EMPA-REG RENAL,2014	NCT01164501	Multinational	374	187	187	64.6 ± 8.9	65.1 ± 8.2	57.2	56.7	45.4 ± 10.2	44.3 ± 10.3	8.02 ± 0.84	8.09 ± 0,80	30.2 ± 5.3	30.3 ± 5.3	N/A	N/A
Kohan,2014	NCT00663260	Multinational	252	168	84	67.0 ± 8.3	67 ± 8.6	66.1	63.1	43.88 ± 10.1	45.84 ± 11.23	8.26 ± 1.01	8.53 ± 1.28	N/A	N/A	17.6 ± 9.6	15.7 ± 9.5
Yale, 2014	NCT01064414	Multinational	269	179	90	68.7± 8.2	68.2± 8.4	59.2	63.3	39.1 ± 6.9	40.1 ± 6.8	7.9 ± 0.9	8.0 ± 0.9	32.9 ± 6.02	33.1 ± 6.5	16.3 ± 7.6	16.4 ± 10.1
EMPA-REG OUTCOME,2015	NCT01131676	Multinational	1,819	1,212	607	67.1 ± 7.6	67.1 ± 8.2	67.3	68.9	48.4 ± 8.2	48.6 ± 7.8	8.07 ± 0.86	8.03 ± 0.85	31.0 ± 5.5	30.9 ± 5.4	N/A	N/A
CANVAS,2017	NCT01032629 NCT01989754	Multinational	2,039	1,110	929	67.6 ± 7.8	67.6 ± 7.6	59	57	49.2 ± 7.8	49.0 ± 8.3	8.3 ± 1.0	8.3 ± 0.9	32.1 ± 5.9	32.5 ± 6.2	16.1 ± 8.4	15.7 ± 8.2
DERIVE,2018	NCT02413398	Multinational	321	160	161	66	68	56.9	56.5	53.3 ± 8.7	53.6 ± 10.6	8.33 ± 1.08	8.03 ± 1.08	32.6 ± 4.7	31.6 ± 5.0	14.3 ± 8.1	14.5 ± 8.3
DELIGHT, 2019	NCT02547935	Multinational	293	145	148	64.7 ± 8.6	64.7 ± 8.5	70	71	50.2 ± 13.0	47.7 ± 13.5	8.44 ± 1.0	8.57 ± 1.2	30.19 ± 5.3	30.34 ± 5.6	17.55 ± 7.7	17.71 ± 9.5
Allegretti,2019	NCT02836873	Multinational	312	157	155	69.3 ± 8.4	69.9 ± 8.29	58.6	67.1	45.4 ± 8.6	44.78 ± 8.1	8.01 ± 0.79	7.95 ± 0.81	30.29 ± 6.0	30.1 ± 5.8	15.54 ± 9.9	16.28 ± 9.0
CREDENCE,2019	NCT02065791	Multinational	4,401	2,202	2,199	62.9 ± 9.2	63.2 ± 9.2	65.4	66.7	56.3 ± 18.2	56.0 ± 18.3	8.3 ± 1.3	8.3 ± 1.3	31.4 ± 6.2	31.3 ± 6.2	15.5 ± 8.7	16.0 ± 8.6
DECLARE-TIMI 58,2019	NCT01730534	Multinational	1,265	606	659	N/A	N/A	N/A	N/A	N/A	N/A	N/A	N/A	N/A	N/A	N/A	N/A
VERTIS CV,2020	NCT01986881	Multinational	1,776	1,178	598	68.3 ± 7.6	68.0 ± 7.5	63.4	66.1	49.5 ± 7.5	49 ± 7.5	8.2 ± 0.9	8.2 ± 0.9	32.3 ± 5.5	32.6 ± 5.6	15.3 ± 8.7	16.4 ± 9.3
DAPA-CKD,2020	NCT03036150	Multinational	2,906	1,455	1,451	64.1 ± 9.8	64.7 ± 9.5	66	68	44.0± 12.6	43.6± 12.6	7.8 ± 1.7	7.8 ± 1.6	N/A	N/A	N/A	N/A
SCORED,2021	NCT03315143	Multicenter	10,584	5,292	5,292	N/A	N/A	55.7	54.5	N/A	N/A	N/A	N/A	N/A	N/A	N/A	N/A

I, intervention; C, control; N/A, not available.

Differences were observed in CKD inclusion criteria across included trials. Cardiovascular outcome trials (CVOTs) mainly defined CKD by eGFR thresholds, while dedicated renal trials used more explicit criteria for UACR and eGFR, with enrichment for proteinuria. The MACE definition was generally consistent, except in FIGARO-DKD and FIDELIO-DKD, which included acute heart failure in the composite outcome. Renal outcome definitions varied slightly but were sufficiently homogeneous for pooled analysis. Detailed definitions are shown in **Table 2**.

**Table 2:** Definition of terms in included studies.

Trial,year	Mean duration of follow-up	Patients enrolled in trials	Patients included Setting in this study	CKD definition (UACR mg/g, eGFR, mL/min/1.73 m²	Drug, Dose, mg/day	Definitions of primary composite cardiovascular outcome	Definitions of renal outcome among included trials in patients with T2DM and CKD
FIDELIO-DKD,2020	2.6 years	T2DM and CKD	T2DM and CKD	UACR 30–300, eGFR 25 to 60 or UACR 300–5,000, eGFR 25 to 75	Finerenone 10/20	CV death, nonfatal MI, nonfatal stroke, HHF	kidney failure, ≥40% sustained eGFR decline, renal death.
FIGARO-DKD,2021	3.4 years	T2DM and CKD	T2DM and CKD	UCAR 30–300, eGFR 25 to 90 or UCAR 300–5,000, eGFR>60	Finerenone 10/20	CV death, nonfatal MI, nonfatal stroke, HHF	Kidney failure,≥40% sustained eGFR decline, renal death
LEADER,2016	3.8 years	T2DM	T2DM and CKD	eGFR <60	Liraglutide titrated to 1.8	CV death, nonfatal MI, nonfatal stroke	N/A
SUSTAIN-6,2016	104 weeks	T2DM	T2DM and CKD	eGFR <60	Semaglutide 0.5/1.0 weekly	CV death, nonfatal MI, nonfatal stroke	N/A
EXSCEL,2017	3.2 years	T2DM	T2DM and CKD	eGFR 30 to 60	exenatide 2 once-weekly	CV death, nonfatal MI, nonfatal stroke	N/A
REWIND,2019	5.4 years	T2DM	T2DM and CKD	eGFR <60	Dulaglutide 1.5 once-weekly	N/A	macroalbuminuria,≥30% sustained eGFR decline, RRT
PIONEER 6,2019	15.9 months	T2DM	T2DM and CKD	eGFR <60	oral Semaglutide 14	CV death, nonfatal MI, nonfatal stroke	N/A
FLOW,2024	3.4 years	T2DM and CKD	T2DM and CKD	UACR 100–<5,000,eGFR 25–75 or UACR>300–<5,000,eGFR 50–75	Semaglutide 1.0 once weekly	CV death, nonfatal MI, nonfatal stroke	persistent eGFR <15 ml/min/1.73 m²,≥50% sustained eGFR decline, RRT, renal death.
EMPA-REG RENAL,2014	52 weeks	T2DM and CKD	T2DM and CKD	eGFR 30 to 60	Empagliflozin 25	N/A	N/A
Kohan,2014	104 weeks	T2DM and CKD	T2DM and CKD	eGFR 30 to 59	Dapagliflozin 5/10	N/A	N/A
Yale, 2014	52 weeks	T2DM and CKD	T2DM and CKD	eGFR 30 to 50	Canagliflozin 100/300	N/A	N/A
EMPA-REG OUTCOME,2015	3.1 years	T2DM	T2DM and CKD	eGFR <60	Empagliflozin 10/25	CV death, nonfatal MI, nonfatal stroke	N/A
CANVAS,2017	188.2 weeks	T2DM	T2DM and CKD	eGFR <60	Canagliflozin 100	CV death, nonfatal MI, nonfatal stroke	ESKD, doubling of SCr, renal death
DERIVE,2018	24 weeks	T2DM and CKD	T2DM and CKD	eGFR 45 to 59	Dapagliflozin 10	N/A	N/A
DELIGHT, 2019	24 weeks	T2DM and CKD	T2DM and CKD	eGFR 25 to 75	Dapagliflozin 10	N/A	N/A
Allegretti,2019	24 weeks	T2DM and CKD	T2DM and CKD	eGFR 30 to 59	Bexagliflozin 20	N/A	N/A
CREDENCE,2019	2.62 years	T2DM and CKD	T2DM and CKD	eGFR 30 to 90	Canagliflozin 100	CV death, nonfatal MI, nonfatal stroke	ESKD, doubling of SCr, renal death
DECLARE-TIMI 58,2019	4.2 years	T2DM	T2DM and CKD	eGFR <60	Dapagliflozin 10	CV death, nonfatal MI, nonfatal stroke	ESKD,≥40% sustained eGFR decline, renal death
VERTIS CV,2020	3.5 years	T2DM	T2DM and CKD	eGFR 30 to 60	Ertugliflozin 5/15	CV death, nonfatal MI, nonfatal stroke	N/A
DAPA-CKD,2020	2.4 years	CKD with or without T2DM	T2DM and CKD	eGFR 25–75	Dapagliflozin 10	N/A	ESKD,≥50% sustained eGFR decline, renal death
SCORED,2021	16.0 months	T2DM and CKD	T2DM and CKD	eGFR 25–60	Sotagliflozin 200 (increased to 400 if tolerated)	CV death, nonfatal MI, nonfatal stroke	eGFR <15 mL/min/1.73 m²,≥50% decline in eGFR, long-term dialysis, RRT

Although baseline UACR and follow-up duration varied across studies, other key potential effect modifiers—including age, sex, baseline eGFR, and HbA1c—were generally comparable across treatment comparisons, providing no clear evidence of a major violation of the transitivity assumption (**Supplementary Table 1**).

### Risk of bias assessment

**Figure 2:**
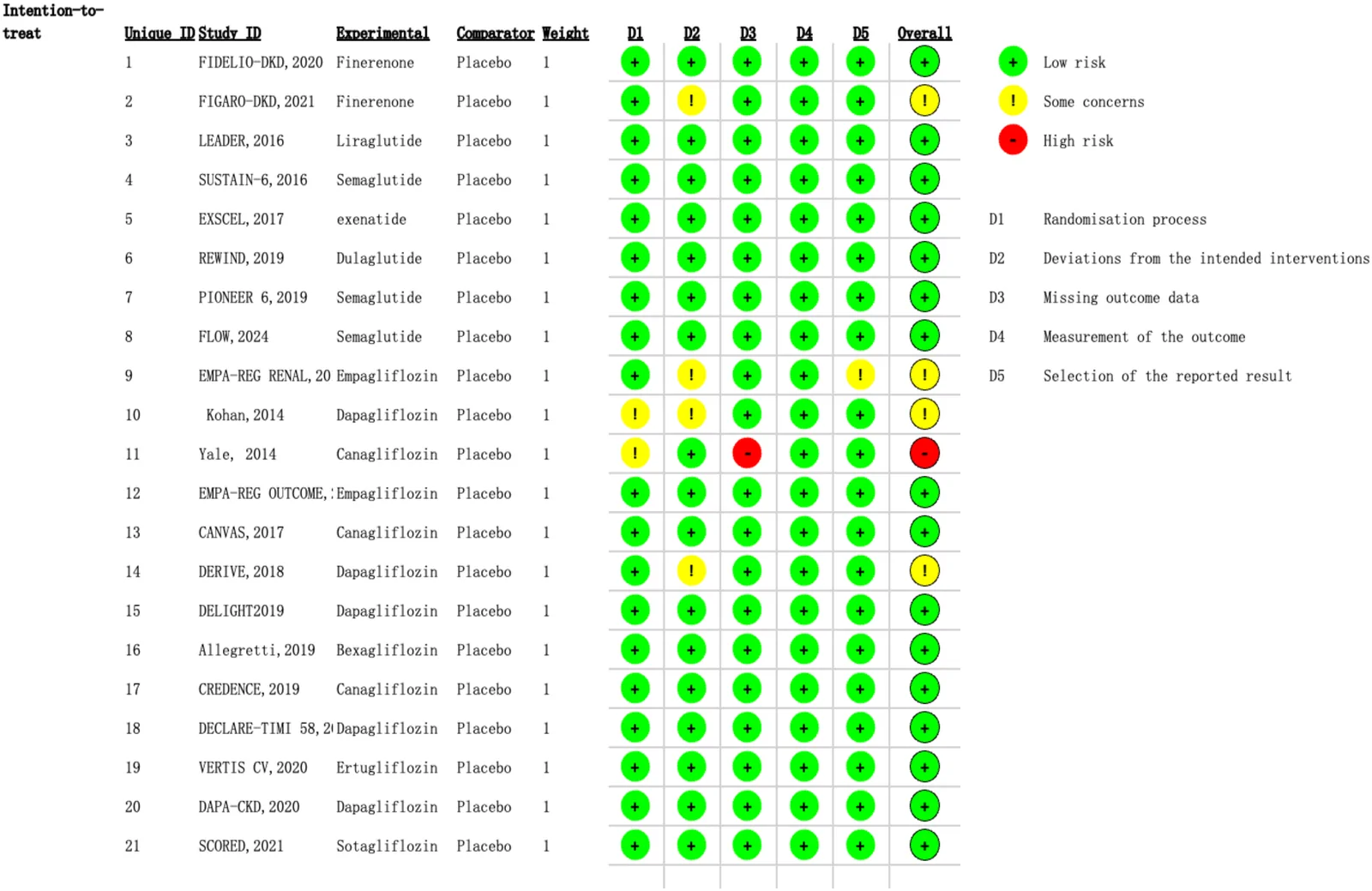
ROB 2.0 risk bias assessment chart.

According to Cochrane RoB 2.0 (**Figure 2**), overall methodological quality of the 21 RCTs was high. In total, 76% (16/21) had low risk of bias, 19% (4/21) had some concerns, and 5% (1/21) were high risk (**Supplementary Figure 1**; **Supplementary Document 3**). “Some concerns” mainly stemmed from randomization (Domain 1) and deviations from intended interventions (Domain 2). Yale (13) was the only study with high risk, due to missing outcome data (Domain 3). Most studies had low risk for outcome measurement (Domain 4) and selective reporting (Domain 5), indicating that the risk of bias related to outcome measurement and reporting was generally limited for key outcomes.

### Network geometry

**Figure 3:**
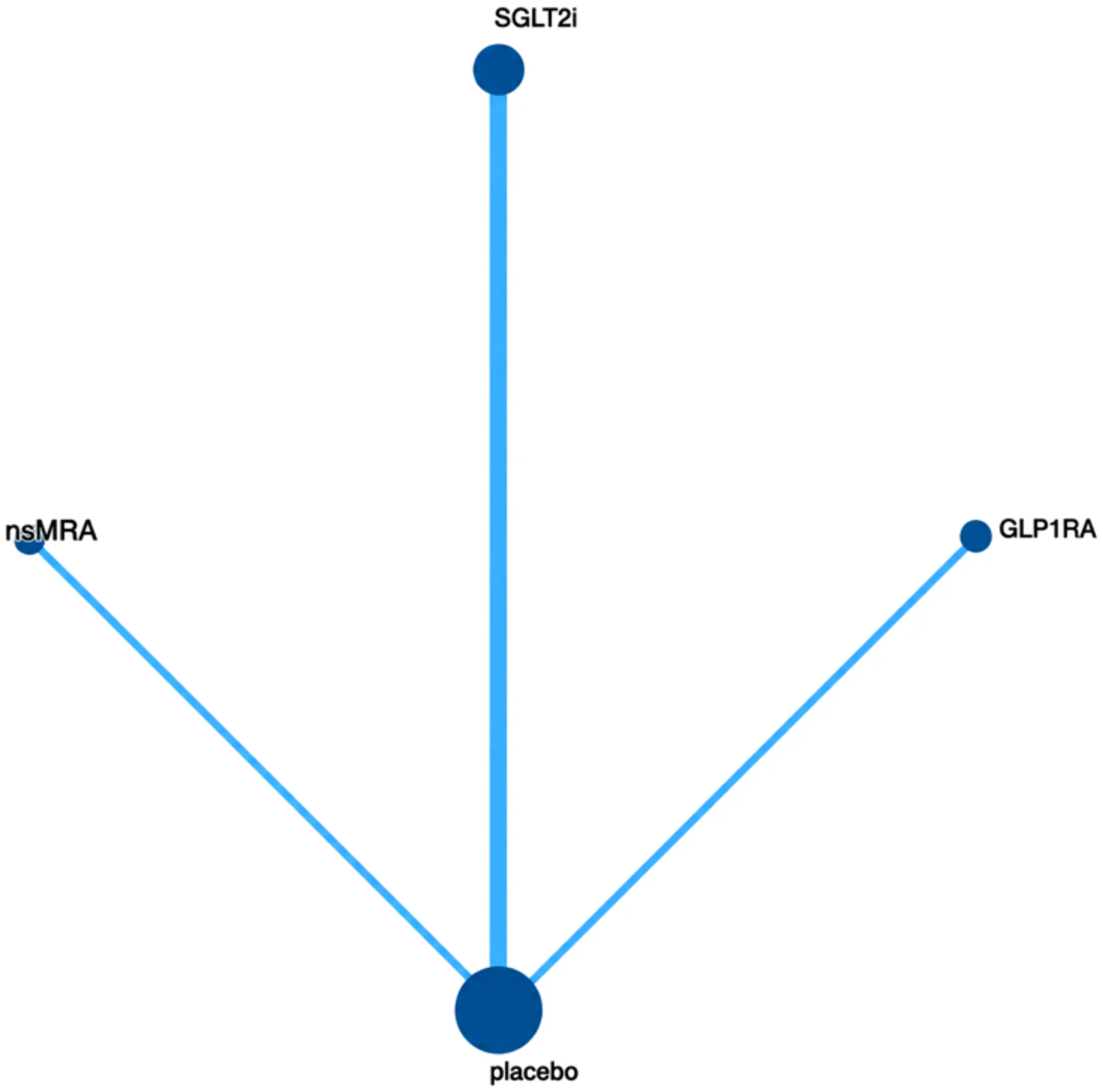
Network plot for MACE.

**Figure 4:**
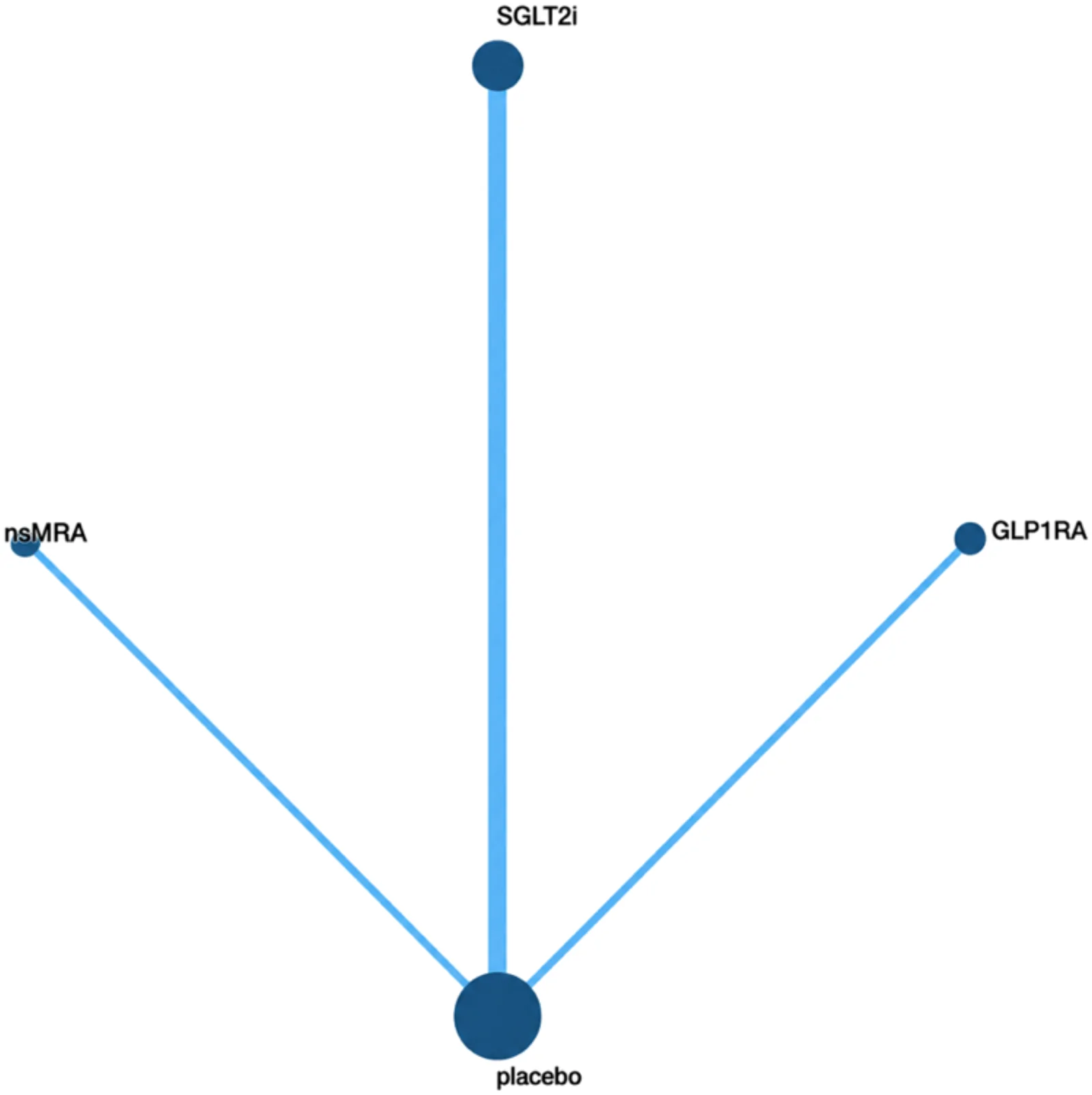
Network plot for major kidney composite outcome.

All five outcome networks shared a star-shaped structure centered on placebo, with no direct head-to-head comparisons among nsMRAs, SGLT2is, and GLP-1RAs. All between-class comparisons were therefore derived from indirect evidence, and no closed loops formed; consistency/inconsistency testing was not applicable (**Figures 3**, **4**; **Supplementary Figures S1**–**S3**).

Evidence strength varied by outcome. For MACE and major kidney outcomes, SGLT2is and GLP-1RAs provided the most evidence, while nsMRAs contributed little. For cardiovascular and all-cause death, SGLT2is dominated, with limited evidence from GLP-1RAs and nsMRAs, increasing uncertainty for mortality endpoints. For HHF, SGLT2is were the main contributor, with minimal input from the other two classes. These network characteristics are important for interpreting ranking results and evidence certainty

### UACR, urinary albumin-to-creatinine ratio; N/A, not available; ESRD, end-stage renal disease.Main outcomes

Eligible data were available from 13 RCTs for MACE, nine RCTs for the major kidney composite outcome, seven RCTs for HHF, nine RCTs for all-cause mortality, and 10 RCTs for cardiovascular death. The individual trials contributing to each outcome network are listed in **Supplementary Table 4**.

### MACE

**Figure 5:**
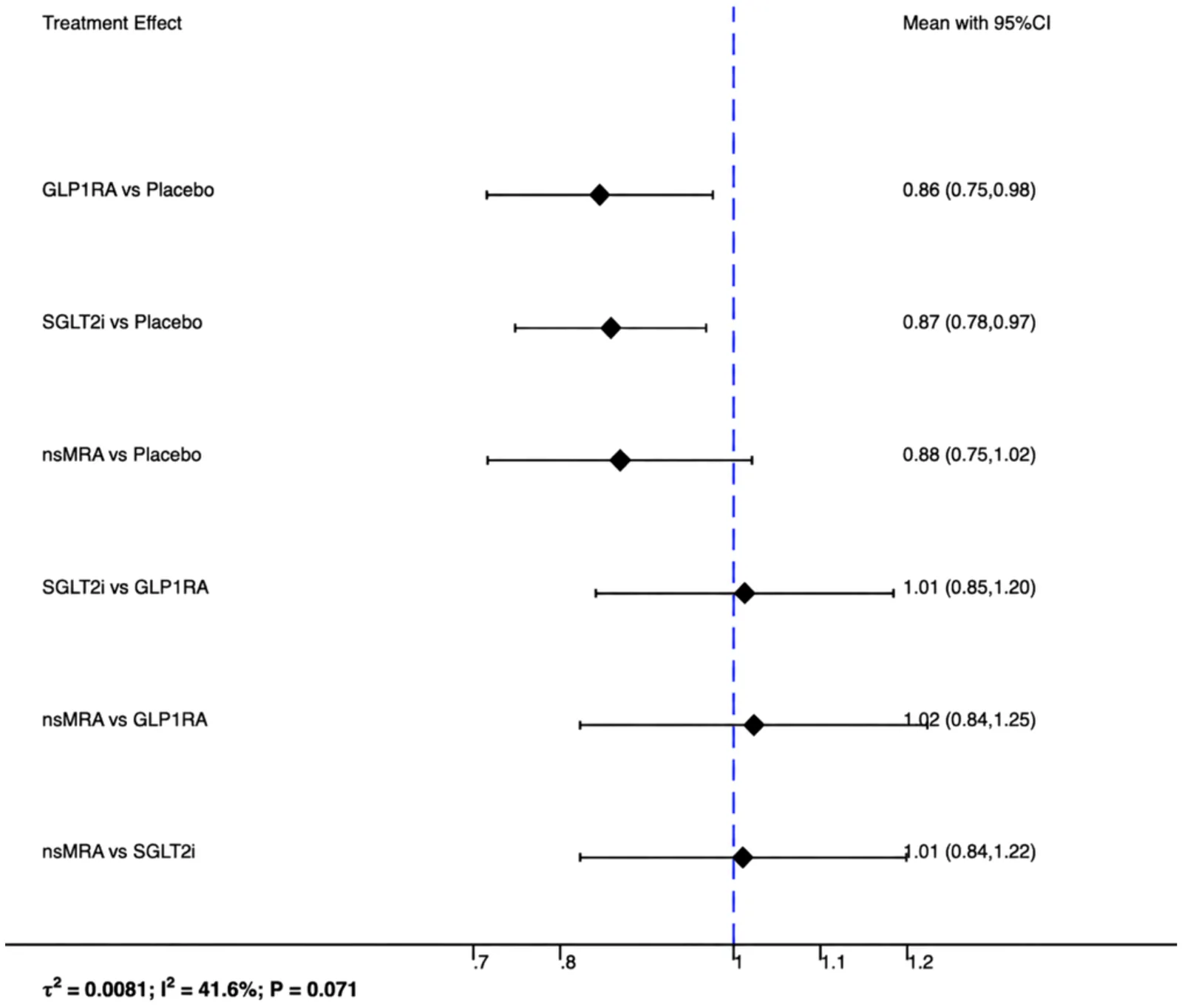
Network meta-analysis reporting RR for MACE in patients with T2DM and CKD.

For MACE, GLP-1RAs and SGLT2is were associated with lower risks than placebo (GLP-1RAs: RR = 0.86, 95% CI 0.75–0.98; SGLT2is: RR = 0.87, 95% CI 0.78–0.97), whereas the estimate for nsMRAs was less precise and its 95% CI included the null (RR = 0.88, 95% CI 0.75–1.02; **Figure 5**). The indirect estimates were RR = 1.01 (95% CI 0.85–1.20) for SGLT2is versus GLP-1RAs, RR = 1.02 (95% CI 0.84–1.25) for nsMRAs versus GLP-1RAs, and RR = 1.01 (95% CI 0.84–1.22) for nsMRAs versus SGLT2is. Taken together, GLP-1RAs and SGLT2is were associated with lower MACE risk than placebo, whereas the estimate for nsMRAs remained inconclusive; however, the indirect evidence did not distinguish among the three active drug classes. Between-study heterogeneity was moderate (τ² = 0.0081, I² = 41.6%, P = 0.071).

### Major kidney composite outcome

**Figure 6:**
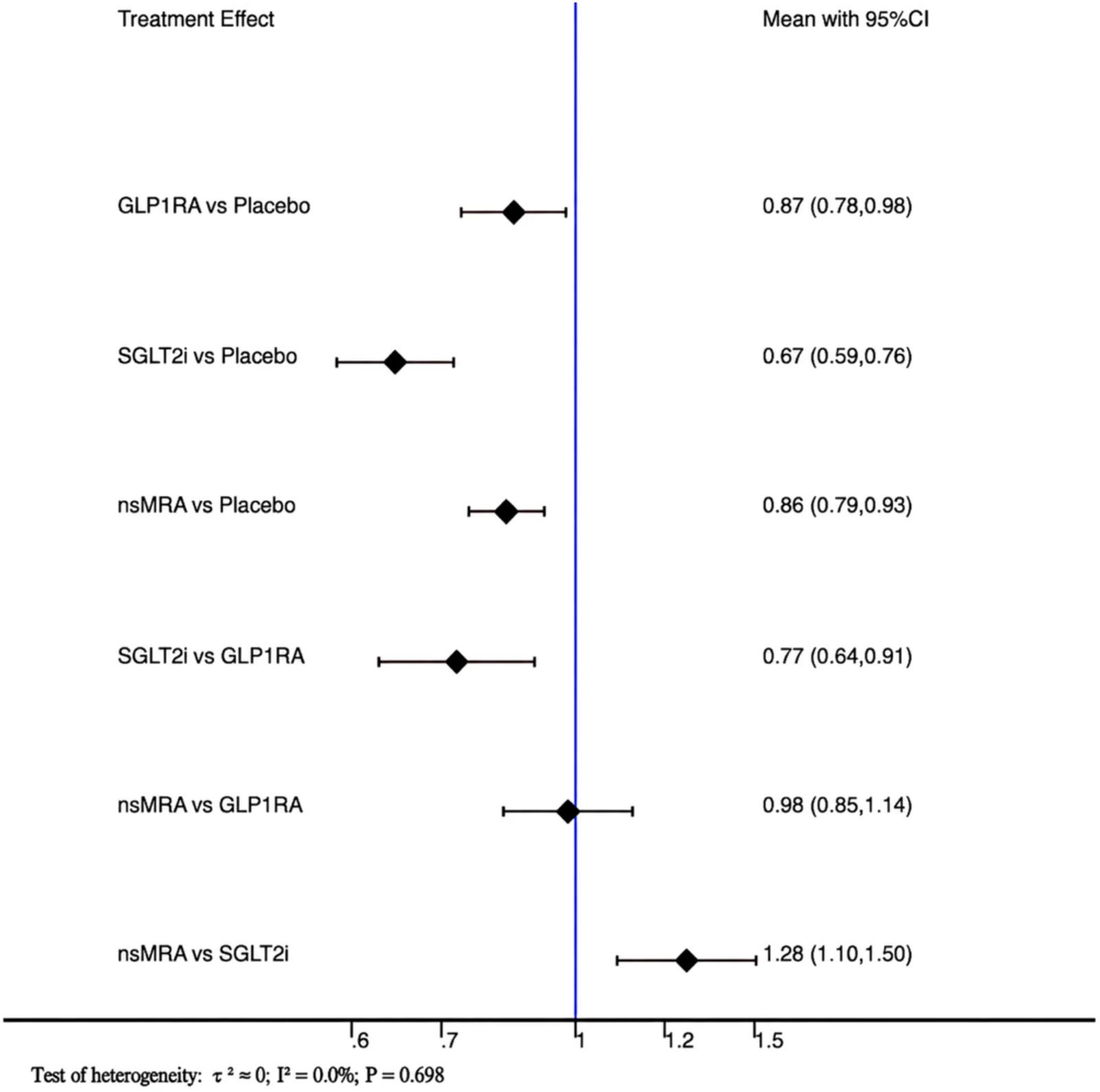
Network meta-analysis reporting RR for major kidney composite outcome in patients with T2DM and CKD.

For the major kidney composite outcome, the random-effects network meta-analysis (**Figure 6**) showed lower risks with all three drug classes than with placebo: SGLT2is (RR = 0.67, 95% CI 0.59–0.76), GLP-1RAs (RR = 0.87, 95% CI 0.78–0.98), and nsMRAs (RR = 0.86, 95% CI 0.79–0.93). The indirect estimates were RR = 0.77 (95% CI 0.64–0.91) for SGLT2is versus GLP-1RAs, RR = 1.28 (95% CI 1.10–1.50) for nsMRAs versus SGLT2is, and RR = 0.98 (95% CI 0.85–1.14) for nsMRAs versus GLP-1RAs. Taken together, all three drug classes were associated with a lower risk of major kidney events than placebo, while the indirect estimates suggested a greater risk reduction with SGLT2is than with GLP-1RAs or nsMRAs; no clear difference was identified between GLP-1RAs and nsMRAs. Estimated between-study heterogeneity was negligible (τ² ≈ 0, I² = 0.0%, P = 0.698).

### HHF

**Figure 7:**
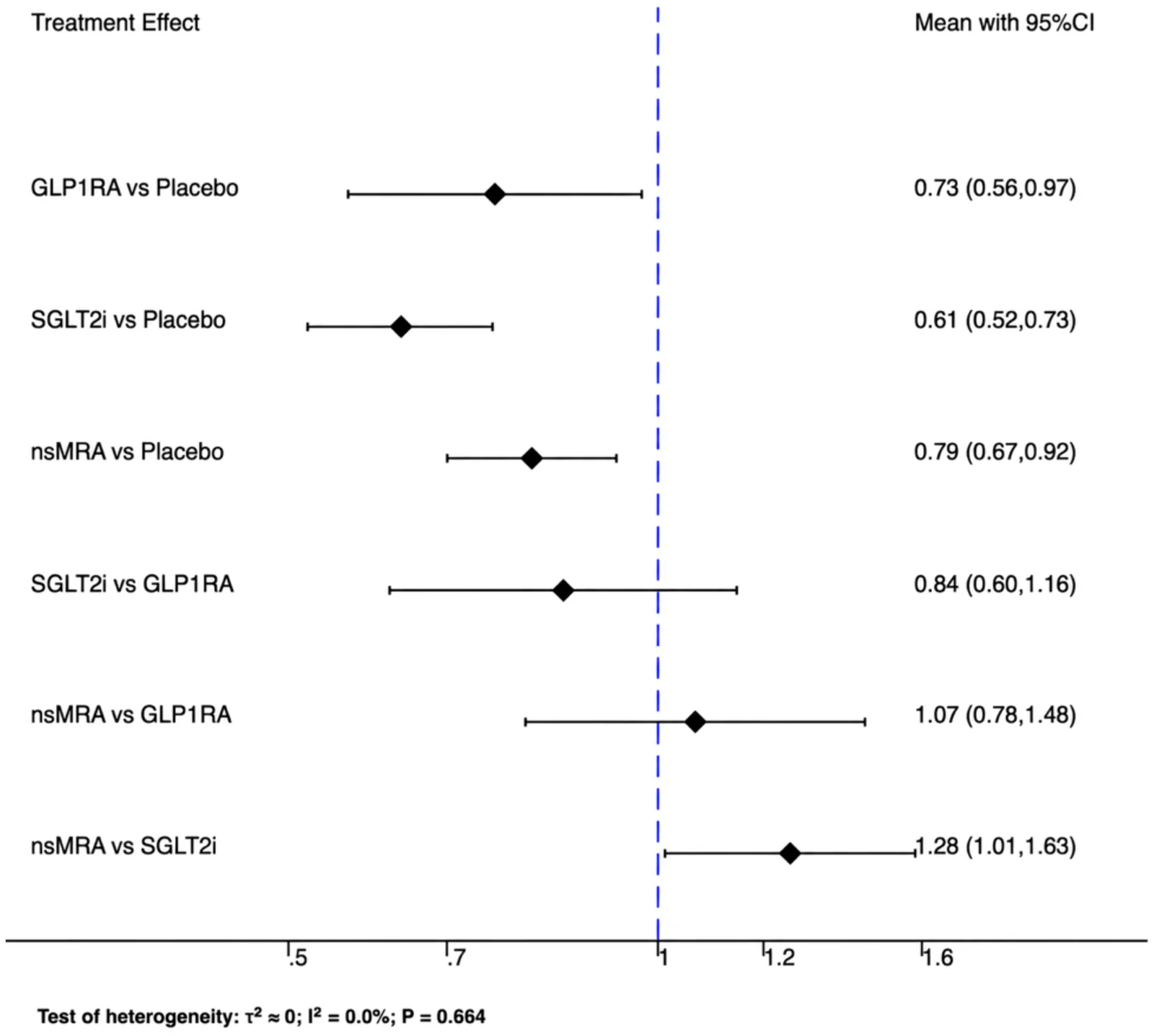
Network meta-analysis reporting RR for HHF in patients with T2DM and CKD.

For hospitalization for heart failure (HHF), the random-effects network meta-analysis (**Figure 7**) showed statistically significant risk reductions versus placebo with GLP-1RAs (RR = 0.73, 95% CI 0.56–0.97), SGLT2is (RR = 0.61, 95% CI 0.52–0.73), and nsMRAs (RR = 0.79, 95% CI 0.67–0.92). Among the active drug classes, the indirect estimate for nsMRAs versus SGLT2is was RR = 1.28 (95% CI 1.01–1.63). The indirect estimates did not show statistically significant differences between SGLT2is and GLP-1RAs (RR = 0.84, 95% CI 0.60–1.16) or between nsMRAs and GLP-1RAs (RR = 1.07, 95% CI 0.78–1.48). Estimated between-study heterogeneity was negligible (τ² ≈ 0, I² = 0.0%, P = 0.664). Taken together, all three drug classes were associated with a lower risk of HHF than placebo, while the indirect estimates suggested a more favorable effect for SGLT2is than for nsMRAs and did not distinguish the other active drug classes.

### All-cause death

**Figure 8:**
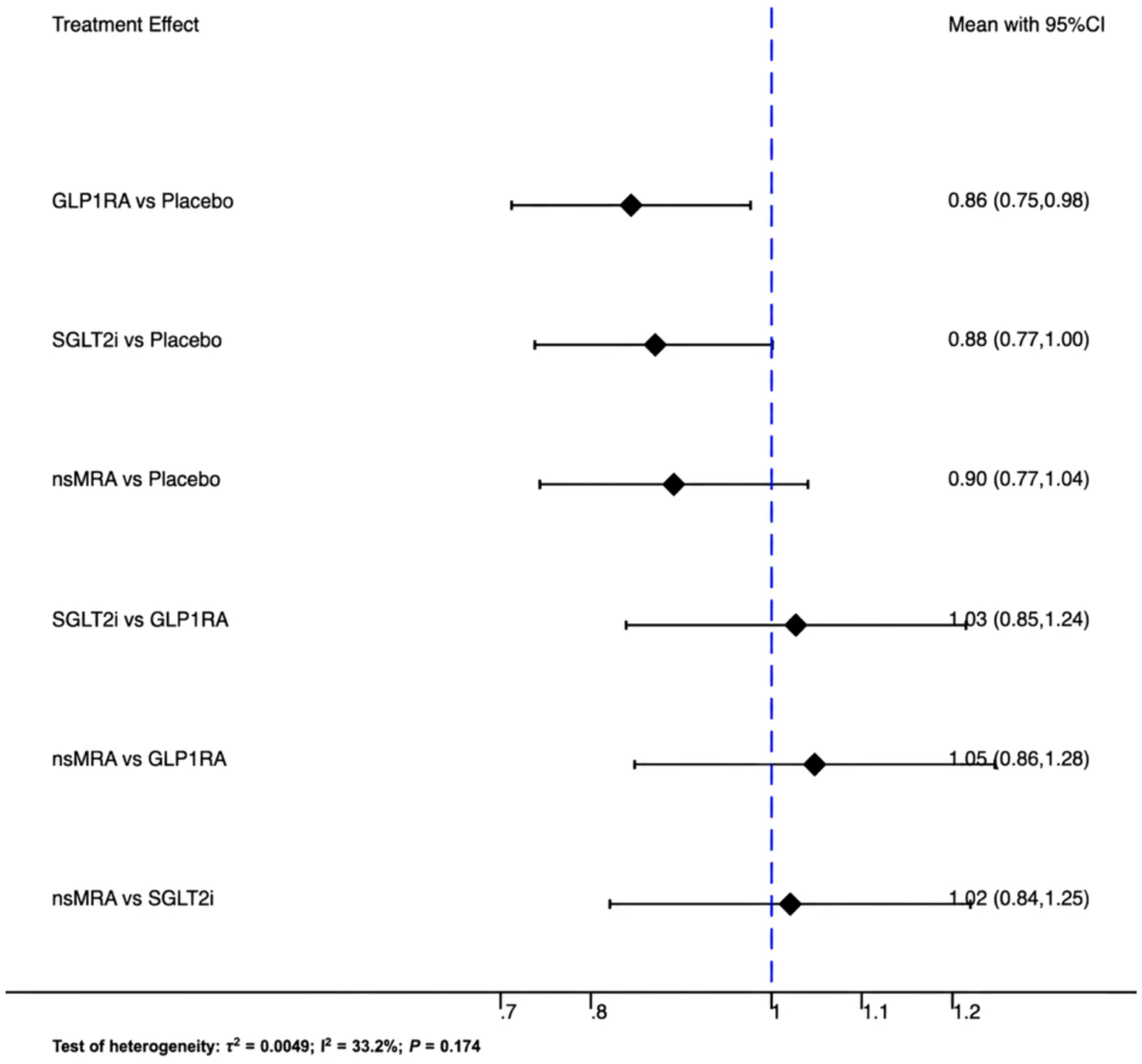
Network meta-analysis reporting RR for ACD with T2DM and CKD.

For all-cause mortality, the random-effects network meta-analysis (**Figure 8**) showed a statistically significant risk reduction with GLP-1RAs versus placebo (RR = 0.86, 95% CI 0.75–0.98). The estimates for SGLT2is (RR = 0.88, 95% CI 0.77–1.00) and nsMRAs (RR = 0.90, 95% CI 0.77–1.04) did not provide clear evidence of a difference from placebo. The indirect estimates did not show statistically significant differences between the active drug classes: SGLT2is versus GLP-1RAs (RR = 1.03, 95% CI 0.85–1.24), nsMRAs versus GLP-1RAs (RR = 1.05, 95% CI 0.86–1.28), and nsMRAs versus SGLT2is (RR = 1.02, 95% CI 0.84–1.25). Between-study heterogeneity was low to moderate (τ² = 0.0049, I² = 33.2%, P = 0.174). Taken together, GLP-1RAs were associated with a lower risk of all-cause mortality than placebo, whereas the evidence for SGLT2is and nsMRAs remained inconclusive; the indirect evidence did not distinguish among the three active drug classes.

### CV death

**Figure 9:**
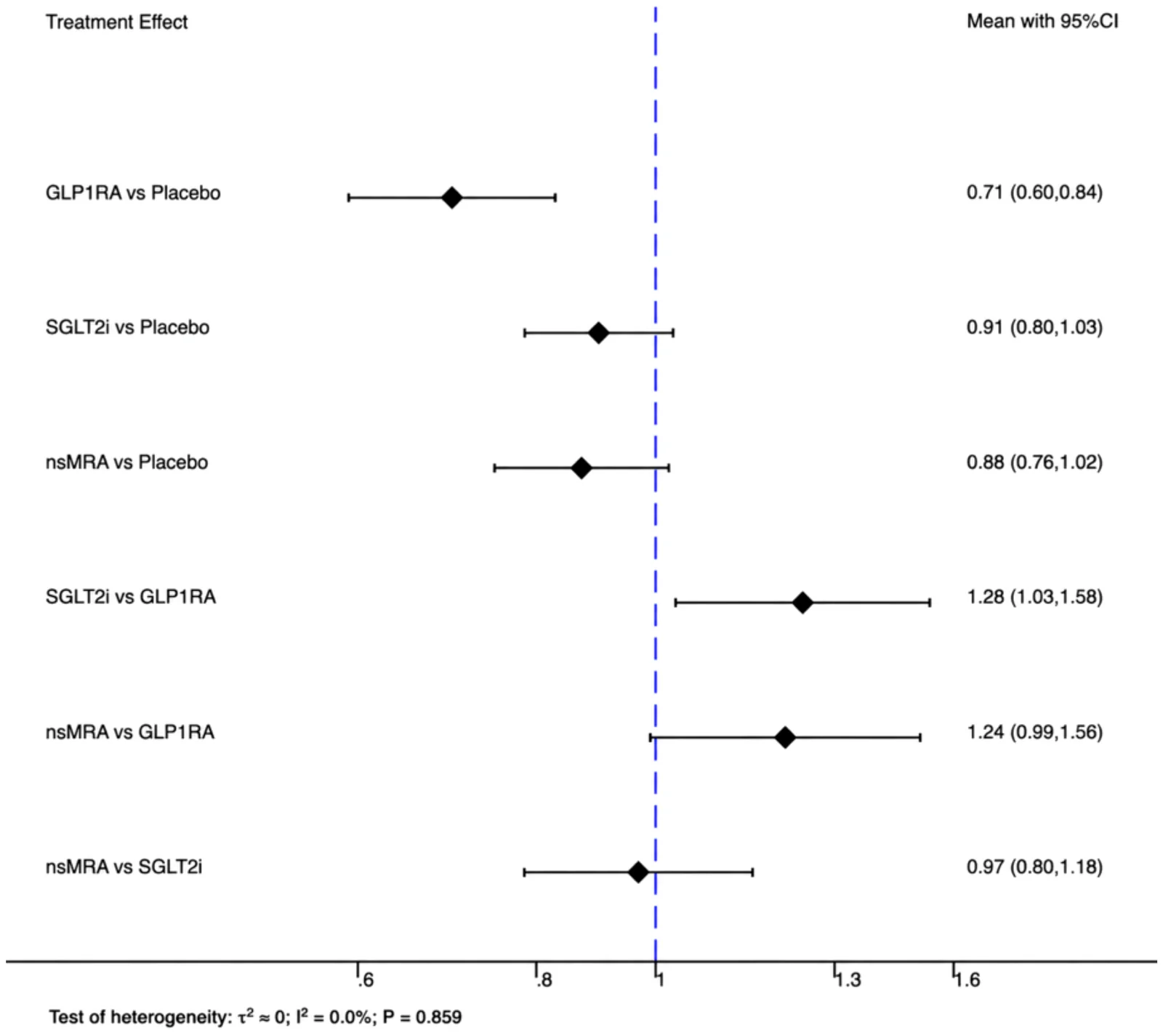
Network meta-analysis reporting RR for CVD in patients with T2DM and CKD.

For cardiovascular death, the random-effects network meta-analysis (**Figure 9**) showed a statistically significant risk reduction with GLP-1RAs versus placebo (RR = 0.71, 95% CI 0.60–0.84). The estimates for SGLT2is (RR = 0.91, 95% CI 0.80–1.03) and nsMRAs (RR = 0.88, 95% CI 0.76–1.02) did not provide clear evidence of a difference from placebo. Among the active drug classes, the indirect estimate for SGLT2is versus GLP-1RAs was RR = 1.28 (95% CI 1.03–1.58). The indirect estimates did not show statistically significant differences between nsMRAs and GLP-1RAs (RR = 1.24, 95% CI 0.99–1.56) or between nsMRAs and SGLT2is (RR = 0.97, 95% CI 0.80–1.18). Estimated between-study heterogeneity was negligible (τ² ≈ 0, I² = 0.0%, P = 0.859). Taken together, GLP-1RAs were associated with a lower risk of cardiovascular death than placebo, and the indirect evidence suggested a more favorable effect estimate for GLP-1RAs than for SGLT2is; the remaining comparisons did not provide clear evidence of between-class differences.

SUCRA values and corresponding rankings for the five main outcomes are summarized in **Supplementary Table 2**, while complete pairwise network estimates are presented in the combined league table (**Supplementary Table 3**). Because the network was star-shaped and contained no head-to-head comparisons among the active drug classes, all between-class estimates were based exclusively on indirect evidence. Accordingly, both the between-class comparisons and treatment rankings should be interpreted cautiously.

### CINeMA assessment of evidence certainty

CINeMA assessment showed low certainty of evidence for most outcomes, with only some comparisons for cardiovascular death reaching moderate certainty (**Supplementary Figures S4**–**S8**; summary grading in **Table 3**).

**Table 3:** CINeMA evaluation result.

Comparison	Number of studies	Within-study bias	Reporting bias	Indirectness	Imprecision	Heterogeneity	Incoherence	Confidence rating
Major adverse cardiovascular events								
GLP-1RA: Placebo	5	No concerns	Low risk	No concerns	No concerns	Some concerns	Some concerns	Low
Placebo: SGLT2i	6	No concerns	Low risk	No concerns	No concerns	Some concerns	Some concerns	Low
nsMRA: Placebo	2	Some concerns	Low risk	Some concerns	Some concerns	No concerns	Some concerns	Very low
GLP-1RA: SGLT2i	0	No concerns	Low risk	No concerns	No concerns	Some concerns	Some concerns	Low
GLP-1RA:nsMRA	0	No concerns	Low risk	No concerns	No concerns	Some concerns	Some concerns	Low
nsMRA: SGLT2i	0	No concerns	Low risk	Some concerns	No concerns	Some concerns	Some concerns	Very low
Major adverse kidney events								
GLP-1RA: Placebo	2	No concerns	Some concerns	Some concerns	No concerns	Some concerns	Some concerns	Very low
Placebo: SGLT2i	5	No concerns	Some concerns	No concerns	No concerns	No concerns	Some concerns	Low
nsMRA: Placebo	2	No concerns	Some concerns	No concerns	No concerns	No concerns	Some concerns	Low
GLP-1RA: SGLT2i	0	No concerns	Some concerns	Some concerns	No concerns	No concerns	Some concerns	Very low
GLP-1RA:nsMRA	0	No concerns	Some concerns	Some concerns	No concerns	No concerns	Some concerns	Very low
nsMRA: SGLT2i	0	No concerns	Some concerns	No concerns	No concerns	No concerns	Some concerns	Low
HHF								
GLP-1RA: Placebo	1	No concerns	Some concerns	No concerns	No concerns	No concerns	Some concerns	Low
Placebo: SGLT2i	4	No concerns	Some concerns	No concerns	No concerns	No concerns	Some concerns	Low
Placebo: nsMRA	2	No concerns	Some concerns	No concerns	No concerns	No concerns	Some concerns	Low
GLP-1RA: SGLT2i	0	No concerns	Some concerns	No concerns	Some concerns	No concerns	Some concerns	Very low
GLP-1RA:nsMRA	0	No concerns	Some concerns	No concerns	Major concerns	No concerns	Some concerns	Very low
SGLT2i:nsMRA	0	No concerns	Some concerns	No concerns	No concerns	No concerns	Some concerns	Low
All cause death								
GLP-1RA: Placebo	3	No concerns	Some concerns	No concerns	No concerns	Some concerns	Some concerns	Very low
Placebo: SGLT2i	4	No concerns	Some concerns	No concerns	Some concerns	No concerns	Some concerns	Very low
nsMRA: Placebo	2	Some concerns	Some concerns	No concerns	Some concerns	No concerns	Some concerns	Very low
GLP-1RA: SGLT2i	0	No concerns	Some concerns	No concerns	Some concerns	Some concerns	Some concerns	Very low
GLP-1RA:nsMRA	0	No concerns	Some concerns	No concerns	Some concerns	Some concerns	Some concerns	Very low
nsMRA: SGLT2i	0	No concerns	Some concerns	No concerns	Some concerns	Some concerns	Some concerns	Very low
CV death								
GLP-1RA: Placebo	2	No concerns	Low risk	No concerns	No concerns	No concerns	Some concerns	Moderate
Placebo: SGLT2i	6	No concerns	Low risk	No concerns	No concerns	No concerns	Some concerns	Moderate
Placebo: nsMRA	2	No concerns	Low risk	No concerns	Some concerns	No concerns	Some concerns	Low
GLP-1RA: SGLT2i	0	No concerns	Low risk	No concerns	No concerns	No concerns	Some concerns	Moderate
GLP-1RA:nsMRA	0	No concerns	Low risk	No concerns	Some concerns	No concerns	Some concerns	Low
SGLT2i:nsMRA	0	No concerns	Low risk	No concerns	No concerns	No concerns	Some concerns	Moderate

Green indicates low risk or no concerns; yellow indicates some concerns; and red indicates major concerns.

### Exploratory metabolic outcomes

Exploratory metabolic outcomes are shown in **Supplementary Figures S9**–**S11**. SGLT2is significantly reduced body weight, HbA1c, and SBP, with low-to-moderate heterogeneity. GLP-1RAs showed beneficial trends but with greater between-study heterogeneity, especially for weight and HbA1c. Details are provided in the **Supplementary Materials**.

### Sensitivity analyses

Sensitivity analyses were conducted according to the source of the included outcome data—CKD-specific trials or CKD subgroup data derived from CVOTs—and differences in kidney outcome definitions (**Supplementary Figures S12**–**S17**). For MACE, the available class-level estimates from both sources were broadly consistent with the main analysis. For the major kidney composite outcome, estimates from CKD-specific trials were consistent with the main analysis, whereas those derived from CVOT subgroup data showed similar directions but wider confidence intervals. Sensitivity analyses examining differences in kidney outcome definitions also yielded results broadly consistent with the main analysis. Full results are provided in the **Supplementary Materials**. Overall, the direction and general pattern of the available estimates were preserved across analyses based on different data sources and kidney outcome definitions, supporting the robustness of the main findings. Trial-specific definitions of the major kidney composite outcome and their eligibility for the endpoint-definition sensitivity analyses are summarized in **Supplementary Table 5**.

### Additional analyses: drug-level comparisons

To complement the class-level analyses and explore potential within-class differences, treatment nodes were disaggregated by individual agent. Drug-level network meta-analyses were conducted for MACE, the major kidney composite outcome, HHF, all-cause mortality, and cardiovascular death. Complete pairwise RR estimates with 95% CIs are presented in **Supplementary Tables S6**–**S10**, respectively. Because no head-to-head trials directly compared the active agents, between-agent estimates were based on indirect evidence and should be interpreted cautiously.

### Safety outcomes

Safety analyses (**Supplementary Figures S18**–**S23**) demonstrated that all three drug classes significantly reduced SAEs versus placebo, with no between-class differences. SGLT2is also decreased any AEs and hyperkalemia but increased genital infections. nsMRAs increased hyperkalemia and showed a trend toward higher discontinuation. No significant differences were seen for severe hypoglycemia. Full results are provided in the **Supplementary Materials**.

## Discussion

This systematic review and network meta-analysis incorporated the latest clinical evidence to comprehensively compare the efficacy and safety of SGLT2is, GLP-1RAs, and nsMRAs on cardiorenal hard endpoints in patients with T2DM and CKD, together with exploratory metabolic profiles including body weight, HbA1c, and SBP.

Compared with placebo, the three drug classes were associated with lower risks of several cardiorenal outcomes, although the observed patterns differed across endpoints. For some outcomes, the indirect estimates suggested more favorable effects for SGLT2is on the major kidney composite outcome and HHF and for GLP-1RAs on cardiovascular death. No statistically significant between-class differences were identified for MACE or all-cause mortality. Because the network was star-shaped and contained no head-to-head comparisons among the active drug classes, all between-class estimates were derived indirectly through placebo and should not be interpreted as establishing a definitive treatment hierarchy.

Our results were generally consistent with previous network meta-analyses (9) focusing on T2D-CKD populations, which also confirmed the prominent renal and cardiorenal benefits of SGLT2is. By incorporating recently published evidence, including the FLOW trial, the present analysis further showed that GLP-1RAs were associated with lower risks of the major kidney composite outcome and HHF than placebo. For cardiovascular death, the indirect estimate suggested a more favorable effect for GLP-1RAs than for SGLT2is, whereas the comparison between GLP-1RAs and nsMRAs did not reach statistical significance. These findings extend the comparative evidence provided by earlier analyses (9, 43, 44), although the between-class estimates remain based exclusively on indirect evidence.

Metabolic parameters are closely linked to CKD progression and cardiovascular risk, thus serving as complementary evaluation indicators. SGLT2is showed stable improvements in weight, glycemic control and blood pressure, whereas GLP-1RA metabolic estimates were more heterogeneous. Such variability may relate to dosage, administration routes, follow-up duration and baseline patient characteristics, suggesting individualized metabolic responses to GLP-1RAs in T2D-CKD.

Notably, the divergent metabolic profiles of the three drug classes partially underpin their distinct cardiorenal protective advantages, which can be further explained by their inherent pharmacological mechanisms. SGLT2is markedly ameliorate glomerular hyperfiltration by modulating proximal tubular glucose–sodium reabsorption and activating tubuloglomerular feedback, thereby lowering intraglomerular pressure, attenuating renal hyperfiltration and reducing proteinuria (45–48). They also improve renal cortical oxygenation, relieve hypoxia-induced tubular injury, and exert prominent anti-inflammatory and antifibrotic effects largely through suppressing NLRP3 inflammasome activation (49, 50).

Unlike SGLT2is, GLP-1RAs exert cardiorenal protection mainly via regulating metabolic and inflammatory pathways. In the kidney, they inhibit sodium–hydrogen exchanger 3, promote natriuresis, and improve glomerular hemodynamics through intrinsic GLP-1R activation (7, 51). They also suppress NF-κB signaling, decrease proinflammatory cytokines, limit macrophage infiltration and AGE-related inflammation, and thereby attenuate tubulointerstitial inflammation and renal fibrosis (52–54). GLP-1RAs also lower atherosclerotic risk and cardiovascular death. Their benefits include indirect metabolic improvement and direct cardioprotective actions, such as endothelial protection, plaque stabilization, anti-apoptosis and NLRP3 inhibition (55–57). Notably, their cardiovascular benefits remain intact independent of weight loss, confirming direct myocardial and vascular protection (58).

nsMRAs counteract excessive mineralocorticoid receptor overactivation to mitigate progressive cardiorenal inflammation and adverse tissue remodeling (59, 60). As a representative nonsteroidal agent, finerenone possesses higher receptor selectivity and better tolerability than traditional steroidal MRAs (61–64), and significantly lowers composite cardiorenal endpoint risks in T2D-CKD patients with heavy proteinuria and progressive renal decline (65, 66).

Each drug class had a distinct safety profile. nsMRAs increased the risk of hyperkalemia, highlighting the need for electrolyte and renal-function monitoring. SGLT2is increased the risk of genital infections but were otherwise generally well tolerated. GLP-1RAs did not increase severe hypoglycemia compared with placebo, consistent with their glucose-dependent mechanism of action. However, these estimates primarily reflect the addition of a single target drug class to background standard care. Evidence regarding the concurrent use of two or all three classes remains sparse, and whether their class-specific adverse effects are additive or modified during combination therapy remains uncertain. The present safety findings should therefore not be extrapolated to dual- or triple-class regimens. Dedicated combination trials and real-world safety studies are needed to address this clinically important evidence gap.

Although age, sex, baseline eGFR, and HbA1c were broadly comparable across the available treatment comparisons, variations in baseline UACR and follow-up duration raise residual uncertainty regarding the transitivity assumption. The design-stratified sensitivity analyses showed no qualitative reversal of the available comparative estimates; however, the CVOT-derived networks were smaller, yielded less precise estimates, and did not include an nsMRA node. Analyses using alternative definitions of the major kidney composite outcome were also broadly consistent with the main analysis. Overall, these sensitivity analyses support the stability of the general comparative patterns but do not eliminate the possibility that differences in trial populations, study design, and outcome definitions influenced the indirect comparisons.

Estimated between-study heterogeneity was low to moderate for MACE and all-cause mortality and negligible for the major kidney composite outcome, hospitalization for heart failure, and cardiovascular death. However, these statistical estimates should be interpreted cautiously because the number of studies contributing to some comparisons was limited. Moreover, low statistical heterogeneity does not exclude residual clinical and methodological heterogeneity related to differences in CKD eligibility criteria, baseline kidney risk, follow-up duration, outcome definitions, and background therapy. The broadly consistent findings of the sensitivity analyses provide some reassurance regarding the general comparative patterns, but do not completely eliminate these sources of uncertainty.

Several limitations should be acknowledged. First, all between-class comparisons were based on indirect evidence through a common placebo comparator, and the network contained no closed loops. Consequently, agreement between direct and indirect evidence could not be evaluated statistically. Differences in trial populations, follow-up duration, outcome definitions, and background therapy may cause the indirect network estimates to differ from effects observed in future head-to-head trials; the relative estimates should therefore be interpreted cautiously. Second, although sensitivity analyses addressing differences in trial design and kidney outcome definitions yielded broadly consistent comparative patterns, the resulting networks were smaller and did not consistently include all drug classes. In particular, the stricter endpoint-definition analysis retained the composite outcomes as originally reported rather than reconstructing standardized endpoints and excluded nsMRA trials because their definitions did not meet the stricter criterion. These limitations reduced the scope and precision of the sensitivity analyses and preclude the complete exclusion of residual bias related to differences in trial design and outcome definitions. Third, the available evidence did not permit evaluation of the safety of concurrent treatment with two or all three target drug classes. Potential additive or interactive adverse effects therefore remain uncertain, limiting the applicability of our safety estimates to combination therapy.

In conclusion, this updated network meta-analysis indicates that SGLT2is, GLP-1RAs, and nsMRAs are associated with improvements in several cardiorenal outcomes in patients with T2D and CKD, with distinct patterns across endpoints. Indirect estimates suggested more favorable effects of SGLT2is on the major kidney composite outcome and HHF, whereas GLP-1RAs showed a more favorable effect on cardiovascular death. These between-class findings should be interpreted cautiously because of the absence of head-to-head comparisons. Treatment selection should therefore be individualized according to cardiovascular and kidney risk, albuminuria, tolerability, and background therapy. Further head-to-head and combination-therapy RCTs using standardized outcome definitions are warranted.

## Data Availability

The original contributions presented in the study are included in the article/**Supplementary Material**. Further inquiries can be directed to the corresponding authors.
