# Supplementary material for: Efficacy and safety of GLP-1RAs, SGLT2is, and nsMRAs for cardiovascular and renal outcomes in T2DM with CKD: a systematic review and network meta-analysis
Source: Front Endocrinol (Lausanne). 2026 Sep 8;17:1937754. doi: 10.3389/fendo.2026.1937754 (PMC13595571; doi:10.3389/fendo.2026.1937754)
Supplement: Supplementary file 1 [file Supplementaryfile1.docx]

**Supplementary materials**

Content

1. Search formulas for different databases.	3

1.1 PubMed	3

1.2 Web of science	5

1.3 Cochrane	6

1.4 EMBASE	7

2.Table S1. Distribution of potential effect modifiers across included trials	10

3.ROB 2.0 Risk Bias Assessment Chart：Detailed risk of bias in each study	13

Domain 1: Risk of bias arising from the randomization process	13

Domain 2: Risk of bias due to deviations from the intended interventions	14

Domain 3: Risk of bias due to missing outcome data	15

Domain 4: Risk of bias in measurement of the outcome	15

Domain 5: Risk of bias in selection of the reported result	16

Overall risk of bias	17

4 Network plot	19

4.1 CV death	19

4.2 All-cause death	19

4.3 HHF	20

5. Treatment rankings and relative-effect estimates	21

5.1 Table S2. SUCRA values for the primary outcomes	21

5.2 Table S3. Combined league table of relative effects for the five main outcomes	21

5.3 Table S4. Contribution of individual trials to the five main outcome networks	23

6. CINeMA Assessment of Evidence Certainty	24

Fig S4.MACE CINeMa Evaluation Result	25

Fig S5. major kidney disease events CINeMa Evaluation Result	26

Fig S6.HHF CINeMa Evaluation Result	26

Fig S7.ACD CINeMa Evaluation Result	27

Fig S8.CVD CINeMa Evaluation Result	27

7 Exploratory Metabolic Outcomes	28

Fig.S9 body weight change forest plot	28

Fig.S10 HbAlc change forest plot	29

Fig.S11 SBP change forest plot	29

8 Sensitivity Analyses	29

8.1 MACE	30

8.2 Major Kidney Composite Outcome	31

9 Additional Analyses: Drug-Level Comparisons	36

Table S5. Drug-level league table for MACE	37

Table S6. Individual Drug League Table-major kidney disease events	38

Table S7. Individual Drug League Table- HHF	38

Table S8. Individual Drug League Table- Death from any cause	38

Table S9. Individual Drug League Table- CV death	39

10 Safety Outcomes	40

Fig.S18 Network meta-analysis reporting RR for SAE in patients with T2DM and CKD	40

Fig.S19 Network meta-analysis reporting RR for Any ae in patients with T2DM and CKD	41

Fig.S20 Network meta-analysis reporting RR for ae leading to discontinuation in patients with T2DM and CKD	42

Fig.S21 Network meta-analysis reporting RR for hyperkalemia in patients with T2DM and CKD	43

Fig.S22 Network meta-analysis reporting RR for severe hypoglycemia in patients with T2DM and CKD	43

Fig.S23 pairwise meta-analysis——Genital Infection	44

# 1. Search formulas for different databases.

## 1.1 PubMed

| #1 | ("Diabetic Nephropathies"[Mesh]) OR ((((((((((((((((((Diabetic Nephropathies[Title/Abstract]) OR (Nephropathies, Diabetic[Title/Abstract])) OR (Nephropathy, Diabetic[Title/Abstract])) OR (Diabetic Kidney Disease[Title/Abstract])) OR (Diabetic Kidney Diseases[Title/Abstract])) OR (Kidney Disease, Diabetic[Title/Abstract])) OR (Kidney Diseases, Diabetic[Title/Abstract])) OR (Diabetic Nephropathy[Title/Abstract])) OR (Diabetic Glomerulosclerosis[Title/Abstract])) OR (Glomerulosclerosis, Diabetic[Title/Abstract])) OR (Intracapillary Glomerulosclerosis[Title/Abstract])) OR (Kimmelstiel-Wilson Disease[Title/Abstract])) OR (Kimmelstiel Wilson Disease[Title/Abstract])) OR (Nodular Glomerulosclerosis[Title/Abstract])) OR (Glomerulosclerosis, Nodular[Title/Abstract])) OR (Kimmelstiel-Wilson Syndrome[Title/Abstract])) OR (Kimmelstiel Wilson Syndrome[Title/Abstract])) OR (Syndrome, Kimmelstiel-Wilson[Title/Abstract])) |
| --- | --- |
| #2 | ("Glucagon-Like Peptide-1 Receptor Agonists"[Mesh] OR "Liraglutide"[Mesh] OR "Exenatide"[Mesh]) OR (((((((((((((((((((((((((((((((((((((((((((((((((((((((((((((((((((((((((((((((((((((((((((((((((((((((((((((((((((((((Glucagon Like Peptide 1 Receptor Agonists[Title/Abstract]) OR (Incretin Mimetics[Title/Abstract])) OR (GLP-1 Analogs[Title/Abstract])) OR (GLP 1 Analogs[Title/Abstract])) OR (GLP-1 Receptor Agonists[Title/Abstract])) OR (GLP 1 Receptor Agonists[Title/Abstract])) OR (liraglutide[Title/Abstract])) OR (Victoza[Title/Abstract])) OR (Saxenda[Title/Abstract])) OR (NN 2211[Title/Abstract])) OR (NN2211[Title/Abstract])) OR (NN-2211[Title/Abstract])) OR (semaglutide[Title/Abstract])) OR (rybelsus[Title/Abstract])) OR (Wegovy[Title/Abstract])) OR (Ozempic[Title/Abstract])) OR (tirzepatide[Title/Abstract])) OR (zepbound[Title/Abstract])) OR (LY3298176[Title/Abstract])) OR (Exenatide[Title/Abstract])) OR (Byetta[Title/Abstract])) OR (AC 2993[Title/Abstract])) OR (Bydureon[Title/Abstract])) OR (Exendin-4[Title/Abstract])) OR (Ex4 Peptide[Title/Abstract])) OR (Peptide, Ex4[Title/Abstract])) OR (Exendin 4[Title/Abstract])) OR (ITCA 650[Title/Abstract])) OR (AC 2993 LAR[Title/Abstract])) OR (glucagon-like peptide 1 (7-36[Title/Abstract]))) OR (beinaglutide[Title/Abstract])) OR (proglucagon (78-107[Title/Abstract]))) OR (GLP-1-(7-36[Title/Abstract]))) OR (dulaglutide[Title/Abstract])) OR (LY 2189265[Title/Abstract])) OR (LY-2189265[Title/Abstract])) OR (LY2189265[Title/Abstract])) OR (Trulicity[Title/Abstract])) OR (rGLP-1 protein[Title/Abstract])) OR (Eperzan[Title/Abstract])) OR (Tanzeum[Title/Abstract])) OR (albiglutide[Title/Abstract])) OR (lixisenatide[Title/Abstract])) OR (DES-38-proline-exendine-4 (Heloderma suspectum)-(1-39)-peptidylpenta-l-lysyl-l-lysinamide[Title/Abstract])) OR (Adlyxin[Title/Abstract])) OR (AQVE-10010[Title/Abstract])) OR (ZP10A peptide[Title/Abstract])) OR (ZP 10[Title/Abstract])) OR (ZP-10[Title/Abstract])) OR (Lyxumia[Title/Abstract])) OR (AVE 010[Title/Abstract])) OR (AVE-010[Title/Abstract])) OR (AVE 0010[Title/Abstract])) OR (AVE0010[Title/Abstract])) OR (AVE-0010[Title/Abstract])) OR (polyethylene glycol loxenatide[Title/Abstract])) OR (loxenatide[Title/Abstract])) OR (PEX168[Title/Abstract])) OR (efpeglenatide[Title/Abstract])) OR (L-Proline, 1-(3-(2-(3-(((5S[Title/Abstract])-5-amino-5-carboxypentyl)amino)propoxy)ethoxy)propyl))) OR (Mazdutide[Title/Abstract])) OR (IBI362[Title/Abstract])) OR (LY3305677[Title/Abstract])) OR (retatrutide[Title/Abstract])) OR (LY3437943[Title/Abstract])) OR (CagriSema[Title/Abstract])) OR (semaglutide[Title/Abstract])) OR (rybelsus[Title/Abstract])) OR (taspoglutide[Title/Abstract])) OR (Xultophy[Title/Abstract])) OR (SAXE[Title/Abstract])) OR (NN-2211[Title/Abstract])) OR (Saxenda[Title/Abstract])) OR (NN-9211[Title/Abstract])) OR (NNC 90-1170[Title/Abstract])) OR (NNC-901170[Title/Abstract])) OR (NN-8022[Title/Abstract])) OR (GLP-1-FC[Title/Abstract])) OR (NN-9931[Title/Abstract])) OR (GLP-fc[Title/Abstract])) OR (NN-9536[Title/Abstract])) OR (NN-9535[Title/Abstract])) OR (NNC-0113-0217[Title/Abstract])) OR (NN9924[Title/Abstract])) OR (Mounjaro[Title/Abstract])) OR (LY-3298176[Title/Abstract])) OR (GSK-716155[Title/Abstract])) OR (Albugon[Title/Abstract])) OR (Syncria[Title/Abstract])) OR (AVE0100[Title/Abstract])) OR (LY-2148568[Title/Abstract])) OR (PGC-GLP-1[Title/Abstract])) OR (PEX-168[Title/Abstract])) OR (des-38-proline-exendine-4[Title/Abstract])) OR (SAR-439977[Title/Abstract])) OR (HM-11260C[Title/Abstract])) OR (LAPS-EXD4[Title/Abstract])) OR (LAPS-Exendin4[Title/Abstract])) OR (LAPSCA-Exendin4[Title/Abstract])) OR (LAPS-exendin 4 analogue[Title/Abstract])) OR (Langlenatide[Title/Abstract])) OR (LAPS-Exendin[Title/Abstract])) OR (pegloxenatide[Title/Abstract])) OR (PEG loxenatide[Title/Abstract])) OR (AC-2993-LAR[Title/Abstract])) OR (LY-3305677[Title/Abstract])) OR (LY2148568[Title/Abstract])) OR (OXM3[Title/Abstract])) OR (OXM-3[Title/Abstract])) OR (AC-2993[Title/Abstract])) OR (IBI-362[Title/Abstract])) OR (EX-4[Title/Abstract])) OR (OWL833[Title/Abstract])) OR (Presendin[Title/Abstract])) OR (PB119[Title/Abstract])) OR (PB-119[Title/Abstract])) OR (PEG-exenatide[Title/Abstract])) OR (LY-3502970[Title/Abstract])) OR (LY3502970[Title/Abstract])) |
| #3 | ("Sodium-Glucose Transporter 2 Inhibitors"[Mesh]) OR (((((((((((((((((((((Sodium Glucose Transporter 2 Inhibitors[Title/Abstract]) OR (SGLT-2 Inhibitors[Title/Abstract])) OR (SGLT 2 Inhibitors[Title/Abstract])) OR (SGLT-2 Inhibitor[Title/Abstract])) OR (Inhibitor, SGLT-2[Title/Abstract])) OR (SGLT 2 Inhibitor[Title/Abstract])) OR (Sodium-Glucose Transporter 2 Inhibitor[Title/Abstract])) OR (Sodium Glucose Transporter 2 Inhibitor[Title/Abstract])) OR (SGLT2 Inhibitor[Title/Abstract])) OR (Inhibitor, SGLT2[Title/Abstract])) OR (Gliflozins[Title/Abstract])) OR (Gliflozin[Title/Abstract])) OR (SGLT2 Inhibitors[Title/Abstract])) OR (empagliflozin[Title/Abstract])) OR (canagliflozin[Title/Abstract])) OR (dapagliflozin[Title/Abstract])) OR (Ertugliflozin[Title/Abstract])) OR (Ipragliflozin[Title/Abstract])) OR (Luseogliflozin[Title/Abstract])) OR (Tofogliflozin[Title/Abstract])) OR (Cangrelor[Title/Abstract])) |
| #4 | ((((non-steroid mineralocorticoid receptor antagonists[Title/Abstract]) OR (nonsteroid mineralocorticoid receptor antagonists[Title/Abstract])) OR (finerenone[Title/Abstract])) OR (esaxerenone[Title/Abstract])) OR (apararenone[Title/Abstract]) |
| #5 | ((((("Randomized Controlled Trial" [Publication Type]) OR ("Controlled Clinical Trial" [Publication Type])) OR ("Clinical Trials as Topic"[Mesh])) OR ("Random Allocation"[Mesh])) OR ("Randomized Controlled Trials as Topic"[Mesh])) OR ((((((((Randomized Controlled Trial[Title/Abstract]) OR (Controlled Clinical Trial[Title/Abstract])) OR (Clinical Trials as Topic[Title/Abstract])) OR (random allocation[Title/Abstract])) OR (randomized controlled trials as topic[Title/Abstract])) OR (randomly[Title/Abstract])) OR (random[Title/Abstract])) OR (RCT[Title/Abstract])) |
| #6 | #2 OR #3 OR #4 |
| #7 | #1 AND #5 AND #6 |

## 1.2 Web of science

| #1 | (((((((((((((((((TS=(Diabetic Nephropathies)) OR TS=(Nephropathies, Diabetic)) OR TS=(Nephropathy, Diabetic)) OR TS=(Diabetic Kidney Disease)) OR TS=(Diabetic Kidney Diseases)) OR TS=(Kidney Disease, Diabetic)) OR TS=(Kidney Diseases, Diabetic)) OR TS=(Diabetic Nephropathy)) OR TS=(Diabetic Glomerulosclerosis)) OR TS=(Glomerulosclerosis, Diabetic)) OR TS=(Intracapillary Glomerulosclerosis)) OR TS=(Kimmelstiel-Wilson Disease)) OR TS=(Kimmelstiel Wilson Disease)) OR TS=(Nodular Glomerulosclerosis)) OR TS=(Glomerulosclerosis, Nodular)) OR TS=(Kimmelstiel-Wilson Syndrome)) OR TS=(Kimmelstiel Wilson Syndrome)) OR TS=(Syndrome, Kimmelstiel-Wilson) |
| --- | --- |
| #2 | ((((((((((((((((((((((((((((((((((((((((((((((((((((((((((((((((((((((((((((((((((((((((((((((((((((((((((((((((((((((TS=(Glucagon Like Peptide 1 Receptor Agonists)) OR TS=(Incretin Mimetics)) OR TS=(GLP-1 Analogs)) OR TS=(GLP 1 Analogs)) OR TS=(GLP-1 Receptor Agonists)) OR TS=(GLP 1 Receptor Agonists)) OR TS=(liraglutide)) OR TS=(Victoza)) OR TS=(Saxenda)) OR TS=(NN 2211)) OR TS=(NN2211)) OR TS=(NN-2211)) OR TS=(semaglutide )) OR TS=(rybelsus)) OR TS=(Wegovy)) OR TS=(Ozempic)) OR TS=(tirzepatide)) OR TS=(zepbound)) OR TS=(LY3298176)) OR TS=(Exenatide)) OR TS=(Byetta)) OR TS=(AC 2993)) OR TS=(Bydureon)) OR TS=(Exendin-4)) OR TS=(Ex4 Peptide)) OR TS=(Peptide, Ex4)) OR TS=(Exendin 4)) OR TS=(ITCA 650)) OR TS=(AC 2993 LAR)) OR TS=(glucagon-like peptide 1 (7-36))) OR TS=(beinaglutide)) OR TS=(proglucagon (78-107))) OR TS=(GLP-1-(7-36))) OR TS=(dulaglutide)) OR TS=(LY 2189265)) OR TS=(LY-2189265)) OR TS=(LY2189265)) OR TS=(Trulicity)) OR TS=(rGLP-1 protein)) OR TS=(Eperzan)) OR TS=(Tanzeum)) OR TS=(albiglutide)) OR TS=(lixisenatide)) OR TS=(DES-38-proline-exendine-4 (Heloderma suspectum)-(1-39)-peptidylpenta-l-lysyl-l-lysinamide)) OR TS=(Adlyxin)) OR TS=(AQVE-10010)) OR TS=(ZP10A peptide)) OR TS=(ZP 10)) OR TS=(ZP-10)) OR TS=(Lyxumia)) OR TS=(AVE 010)) OR TS=(AVE-010)) OR TS=(AVE 0010)) OR TS=(AVE0010)) OR TS=(AVE-0010)) OR TS=(polyethylene glycol loxenatide)) OR TS=(loxenatide)) OR TS=(PEX168)) OR TS=(efpeglenatide)) OR TS=(L-Proline, 1-(3-(2-(3-(((5S)-5-amino-5-carboxypentyl)amino)propoxy)ethoxy)propyl))) OR TS=(Mazdutide)) OR TS=(IBI362)) OR TS=(LY3305677)) OR TS=(retatrutide)) OR TS=(LY3437943)) OR TS=(CagriSema)) OR TS=(semaglutide)) OR TS=(rybelsus)) OR TS=(taspoglutide)) OR TS=(Xultophy)) OR TS=(SAXE)) OR TS=(NN-2211)) OR TS=(Saxenda)) OR TS=(NN-9211)) OR TS=(NNC 90-1170)) OR TS=(NNC-901170)) OR TS=(NN-8022)) OR TS=(GLP-1-FC)) OR TS=(NN-9931)) OR TS=(GLP-fc)) OR TS=(NN-9536)) OR TS=(NN-9535)) OR TS=(NNC-0113-0217)) OR TS=(NN9924)) OR TS=(Mounjaro)) OR TS=(LY-3298176)) OR TS=(GSK-716155)) OR TS=(Albugon)) OR TS=(Syncria)) OR TS=(AVE0100)) OR TS=(LY-2148568)) OR TS=(PGC-GLP-1)) OR TS=(PEX-168)) OR TS=(des-38-proline-exendine-4)) OR TS=(SAR-439977)) OR TS=(HM-11260C)) OR TS=(LAPS-EXD4)) OR TS=(LAPS-Exendin4)) OR TS=(LAPSCA-Exendin4)) OR TS=(LAPS-exendin 4 analogue)) OR TS=(Langlenatide)) OR TS=(LAPS-Exendin)) OR TS=(pegloxenatide)) OR TS=(PEG loxenatide)) OR TS=(AC-2993-LAR)) OR TS=(LY-3305677)) OR TS=(LY2148568)) OR TS=(OXM3)) OR TS=(OXM-3)) OR TS=(AC-2993)) OR TS=(IBI-362)) OR TS=(EX-4)) OR TS=(OWL833)) OR TS=(Presendin)) OR TS=(PB119)) OR TS=(PB-119)) OR TS=(PEG-exenatide)) OR TS=(LY-3502970)) OR TS=(LY3502970) |
| #3 | ((((((((((((((((((((TS=(Sodium Glucose Transporter 2 Inhibitors)) OR TS=(SGLT-2 Inhibitors)) OR TS=(SGLT 2 Inhibitors)) OR TS=(SGLT-2 Inhibitor)) OR TS=(Inhibitor, SGLT-2)) OR TS=(SGLT 2 Inhibitor)) OR TS=(Sodium-Glucose Transporter 2 Inhibitor)) OR TS=(Sodium Glucose Transporter 2 Inhibitor)) OR TS=(SGLT2 Inhibitor)) OR TS=(Inhibitor, SGLT2)) OR TS=(Gliflozins)) OR TS=(Gliflozin)) OR TS=(SGLT2 Inhibitors)) OR TS=(empagliflozin )) OR TS=(canagliflozin)) OR TS=(dapagliflozin )) OR TS=(Ertugliflozin)) OR TS=(Ipragliflozin)) OR TS=(Luseogliflozin)) OR TS=(Tofogliflozin)) OR TS=(Cangrelor) |
| #4 | ((((TS=(non-steroid mineralocorticoid receptor antagonists)) OR TS=(nonsteroid mineralocorticoid receptor antagonists)) OR TS=(finerenone)) OR TS=(esaxerenone)) OR TS=(apararenone) |
| #5 | (((((TS=(Randomized Controlled Trial )) OR TS=(Clinical Trials)) OR TS=(random allocation)) OR TS=(randomly)) OR TS=(random)) OR TS=(RCT) |
| #6 | #2 OR #3 OR #4 |
| #7 | #1 AND #5 AND #6 |

## 1.3 Cochrane

| #1 | (Diabetic Nephropathy):ti,ab,kw OR (Nephropathies, Diabetic):ti,ab,kw OR (Nephropathy, Diabetic):ti,ab,kw OR (Diabetic Kidney Disease):ti,ab,kw OR (Diabetic Kidney Diseases):ti,ab,kw OR (Kidney Disease,Diabetic):ti,ab,kw OR (Kidney Diseases,Diabetic):ti,ab,kw OR (Diabetic Nephropathy):ti,ab,kw OR (Diabetic Glomerulosclerosis):ti,ab,kw OR (Glomerulosclerosis, Diabetic):ti,ab,kw OR (Intracapillary Glomerulosclerosis):ti,ab,kw OR (Kimmelstiel-Wilson Disease):ti,ab,kw OR (Kimmelstiel Wilson Disease):ti,ab,kw OR (Nodular Glomerulosclerosis):ti,ab,kw OR (Glomerulosclerosis, Nodular):ti,ab,kw OR (Kimmelstiel-Wilson Syndrome):ti,ab,kw OR (Kimmelstiel Wilson Syndrome):ti,ab,kw OR (Syndrome, Kimmelstiel-Wilson):ti,ab,kw |
| --- | --- |
| #2 | (GLP-1 Analogs):ti,ab,kw OR (GLP 1 Analogs):ti,ab,kw OR (GLP-1 Receptor Agonists):ti,ab,kw OR (GLP 1 Receptor Agonists):ti,ab,kw OR (liraglutide):ti,ab,kw OR (Victoza):ti,ab,kw OR (Saxenda):ti,ab,kw OR (NN 2211):ti,ab,kw OR (NN2211):ti,ab,kw OR (NN-2211):ti,ab,kw OR (semaglutide):ti,ab,kw OR (rybelsus):ti,ab,kw OR (Wegovy):ti,ab,kw OR (Ozempic):ti,ab,kw OR (tirzepatide):ti,ab,kw OR (zepbound):ti,ab,kw OR (LY3298176):ti,ab,kw OR (Exenatide):ti,ab,kw OR (Byetta):ti,ab,kw OR (AC 2993):ti,ab,kw OR (Bydureon):ti,ab,kw OR (Exendin-4):ti,ab,kw OR (Ex4 Peptide):ti,ab,kw OR (Peptide, Ex4):ti,ab,kw OR (Exendin 4):ti,ab,kw OR (ITCA 650):ti,ab,kw OR (AC 2993 LAR):ti,ab,kw OR (beinaglutide):ti,ab,kw OR (dulaglutide):ti,ab,kw OR (LY 2189265):ti,ab,kw OR (LY-2189265):ti,ab,kw OR (LY2189265):ti,ab,kw OR (Trulicity):ti,ab,kw OR (rGLP-1 protein):ti,ab,kw OR (Eperzan):ti,ab,kw OR (Tanzeum):ti,ab,kw OR (albiglutide):ti,ab,kw OR (lixisenatide):ti,ab,kw OR (Adlyxin):ti,ab,kw OR (AQVE-10010):ti,ab,kw OR (ZP10A peptide):ti,ab,kw OR (ZP 10):ti,ab,kw OR (ZP-10):ti,ab,kw OR (Lyxumia):ti,ab,kw OR (AVE 010):ti,ab,kw OR (AVE-010):ti,ab,kw OR (AVE 0010):ti,ab,kw OR (AVE0010):ti,ab,kw OR (AVE-0010):ti,ab,kw OR (polyethylene glycol loxenatide):ti,ab,kw OR (loxenatide):ti,ab,kw OR (PEX168):ti,ab,kw OR (efpeglenatide):ti,ab,kw OR (Mazdutide):ti,ab,kw OR (IBI362):ti,ab,kw OR (LY3305677):ti,ab,kw OR (retatrutide):ti,ab,kw OR (LY3437943):ti,ab,kw OR (CagriSema):ti,ab,kw OR (semaglutide):ti,ab,kw OR (rybelsus):ti,ab,kw OR (taspoglutide):ti,ab,kw OR (Xultophy):ti,ab,kw OR (SAXE):ti,ab,kw OR (NN-2211):ti,ab,kw OR (Saxenda):ti,ab,kw OR (NN-9211):ti,ab,kw OR (NNC-901170):ti,ab,kw OR (NN-8022):ti,ab,kw OR (NN-9931):ti,ab,kw OR (GLP-fc):ti,ab,kw OR (NN-9536):ti,ab,kw OR (NN-9535):ti,ab,kw OR (NN9924):ti,ab,kw OR (Mounjaro):ti,ab,kw OR (LY-3298176):ti,ab,kw OR (GSK-716155):ti,ab,kw OR (Albugon):ti,ab,kw OR (Syncria):ti,ab,kw OR (AVE0100):ti,ab,kw OR (LY-2148568):ti,ab,kw OR (PGC-GLP-1):ti,ab,kw OR (PEX-168):ti,ab,kw OR (SAR-439977):ti,ab,kw OR (HM-11260C):ti,ab,kw OR (LAPS-EXD4):ti,ab,kw OR (LAPS-Exendin4):ti,ab,kw OR (LAPSCA-Exendin4):ti,ab,kw OR (LAPS-exendin 4 analogue):ti,ab,kw OR (Langlenatide):ti,ab,kw OR (LAPS-Exendin):ti,ab,kw OR (pegloxenatide):ti,ab,kw OR (PEG loxenatide):ti,ab,kw OR (LY-3305677):ti,ab,kw OR (LY2148568):ti,ab,kw OR (OXM3):ti,ab,kw OR (OXM-3):ti,ab,kw OR (AC-2993):ti,ab,kw OR (IBI-362):ti,ab,kw OR (EX-4):ti,ab,kw OR (OWL833):ti,ab,kw OR (Presendin):ti,ab,kw OR (PB119):ti,ab,kw OR (PB-119):ti,ab,kw OR (PEG-exenatide):ti,ab,kw OR (LY-3502970):ti,ab,kw OR (LY3502970):ti,ab,kw |
| #3 | (Sodium Glucose Transporter 2 Inhibitors):ti,ab,kw OR (SGLT-2 Inhibitors):ti,ab,kw OR (SGLT 2 Inhibitors):ti,ab,kw OR (SGLT-2 Inhibitor):ti,ab,kw OR (Inhibitor, SGLT-2):ti,ab,kw OR (SGLT 2 Inhibitor):ti,ab,kw OR (Sodium-Glucose Transporter 2 Inhibitor):ti,ab,kw OR (Sodium Glucose Transporter 2 Inhibitor):ti,ab,kw OR (SGLT2 Inhibitor):ti,ab,kw OR (Inhibitor, SGLT2):ti,ab,kw OR (Gliflozins):ti,ab,kw OR (Gliflozin):ti,ab,kw OR (SGLT2 Inhibitors):ti,ab,kw OR (empagliflozin):ti,ab,kw OR (canagliflozin):ti,ab,kw OR (dapagliflozin):ti,ab,kw OR (Ertugliflozin):ti,ab,kw OR (Ipragliflozin):ti,ab,kw OR (Luseogliflozin):ti,ab,kw OR (Tofogliflozin):ti,ab,kw OR (Cangrelor):ti,ab,kw |
| #4 | (non-steroid mineralocorticoid receptor antagonists):ti,ab,kw OR (nonsteroid mineralocorticoid receptor antagonists):ti,ab,kw OR (finerenone):ti,ab,kw OR (esaxerenone):ti,ab,kw OR (apararenone):ti,ab,kw |
| #5 | ("randomized clinical trial"):pt OR ("controlled clinical trial"):pt OR ("clinical trial"):ti,ab,kw OR ("random allocation"):ti,ab,kw OR ("clinical trial"):ti,ab,kw ("randomly"):ti,ab,kw |
| #6 | #2 OR #3 OR #4 |
| #7 | #1 AND # 5 AND #6 |

## 1.4 EMBASE

| #1 | (Diabetic Nephropathy):ti,ab,kw OR (Nephropathies, Diabetic):ti,ab,kw OR (Nephropathy, Diabetic):ti,ab,kw OR (Diabetic Kidney Disease):ti,ab,kw OR (Diabetic Kidney Diseases):ti,ab,kw OR (Kidney Disease,Diabetic):ti,ab,kw OR (Kidney Diseases,Diabetic):ti,ab,kw OR (Diabetic Nephropathy):ti,ab,kw OR (Diabetic Glomerulosclerosis):ti,ab,kw OR (Glomerulosclerosis, Diabetic):ti,ab,kw OR (Intracapillary Glomerulosclerosis):ti,ab,kw OR (Kimmelstiel-Wilson Disease):ti,ab,kw OR (Kimmelstiel Wilson Disease):ti,ab,kw OR (Nodular Glomerulosclerosis):ti,ab,kw OR (Glomerulosclerosis, Nodular):ti,ab,kw OR (Kimmelstiel-Wilson Syndrome):ti,ab,kw OR (Kimmelstiel Wilson Syndrome):ti,ab,kw OR (Syndrome, Kimmelstiel-Wilson):ti,ab,kw |
| --- | --- |
| #2 | (GLP-1 Analogs):ti,ab,kw OR (GLP 1 Analogs):ti,ab,kw OR (GLP-1 Receptor Agonists):ti,ab,kw OR (GLP 1 Receptor Agonists):ti,ab,kw OR (liraglutide):ti,ab,kw OR (Victoza):ti,ab,kw OR (Saxenda):ti,ab,kw OR (NN 2211):ti,ab,kw OR (NN2211):ti,ab,kw OR (NN-2211):ti,ab,kw OR (semaglutide):ti,ab,kw OR (rybelsus):ti,ab,kw OR (Wegovy):ti,ab,kw OR (Ozempic):ti,ab,kw OR (tirzepatide):ti,ab,kw OR (zepbound):ti,ab,kw OR (LY3298176):ti,ab,kw OR (Exenatide):ti,ab,kw OR (Byetta):ti,ab,kw OR (AC 2993):ti,ab,kw OR (Bydureon):ti,ab,kw OR (Exendin-4):ti,ab,kw OR (Ex4 Peptide):ti,ab,kw OR (Peptide, Ex4):ti,ab,kw OR (Exendin 4):ti,ab,kw OR (ITCA 650):ti,ab,kw OR (AC 2993 LAR):ti,ab,kw OR (beinaglutide):ti,ab,kw OR (dulaglutide):ti,ab,kw OR (LY 2189265):ti,ab,kw OR (LY-2189265):ti,ab,kw OR (LY2189265):ti,ab,kw OR (Trulicity):ti,ab,kw OR (rGLP-1 protein):ti,ab,kw OR (Eperzan):ti,ab,kw OR (Tanzeum):ti,ab,kw OR (albiglutide):ti,ab,kw OR (lixisenatide):ti,ab,kw OR (Adlyxin):ti,ab,kw OR (AQVE-10010):ti,ab,kw OR (ZP10A peptide):ti,ab,kw OR (ZP 10):ti,ab,kw OR (ZP-10):ti,ab,kw OR (Lyxumia):ti,ab,kw OR (AVE 010):ti,ab,kw OR (AVE-010):ti,ab,kw OR (AVE 0010):ti,ab,kw OR (AVE0010):ti,ab,kw OR (AVE-0010):ti,ab,kw OR (polyethylene glycol loxenatide):ti,ab,kw OR (loxenatide):ti,ab,kw OR (PEX168):ti,ab,kw OR (efpeglenatide):ti,ab,kw OR (Mazdutide):ti,ab,kw OR (IBI362):ti,ab,kw OR (LY3305677):ti,ab,kw OR (retatrutide):ti,ab,kw OR (LY3437943):ti,ab,kw OR (CagriSema):ti,ab,kw OR (semaglutide):ti,ab,kw OR (rybelsus):ti,ab,kw OR (taspoglutide):ti,ab,kw OR (Xultophy):ti,ab,kw OR (SAXE):ti,ab,kw OR (NN-2211):ti,ab,kw OR (Saxenda):ti,ab,kw OR (NN-9211):ti,ab,kw OR (NNC-901170):ti,ab,kw OR (NN-8022):ti,ab,kw OR (NN-9931):ti,ab,kw OR (GLP-fc):ti,ab,kw OR (NN-9536):ti,ab,kw OR (NN-9535):ti,ab,kw OR (NN9924):ti,ab,kw OR (Mounjaro):ti,ab,kw OR (LY-3298176):ti,ab,kw OR (GSK-716155):ti,ab,kw OR (Albugon):ti,ab,kw OR (Syncria):ti,ab,kw OR (AVE0100):ti,ab,kw OR (LY-2148568):ti,ab,kw OR (PGC-GLP-1):ti,ab,kw OR (PEX-168):ti,ab,kw OR (SAR-439977):ti,ab,kw OR (HM-11260C):ti,ab,kw OR (LAPS-EXD4):ti,ab,kw OR (LAPS-Exendin4):ti,ab,kw OR (LAPSCA-Exendin4):ti,ab,kw OR (LAPS-exendin 4 analogue):ti,ab,kw OR (Langlenatide):ti,ab,kw OR (LAPS-Exendin):ti,ab,kw OR (pegloxenatide):ti,ab,kw OR (PEG loxenatide):ti,ab,kw OR (LY-3305677):ti,ab,kw OR (LY2148568):ti,ab,kw OR (OXM3):ti,ab,kw OR (OXM-3):ti,ab,kw OR (AC-2993):ti,ab,kw OR (IBI-362):ti,ab,kw OR (EX-4):ti,ab,kw OR (OWL833):ti,ab,kw OR (Presendin):ti,ab,kw OR (PB119):ti,ab,kw OR (PB-119):ti,ab,kw OR (PEG-exenatide):ti,ab,kw OR (LY-3502970):ti,ab,kw OR (LY3502970):ti,ab,kw |
| #3 | (Sodium Glucose Transporter 2 Inhibitors):ti,ab,kw OR (SGLT-2 Inhibitors):ti,ab,kw OR (SGLT 2 Inhibitors):ti,ab,kw OR (SGLT-2 Inhibitor):ti,ab,kw OR (Inhibitor, SGLT-2):ti,ab,kw OR (SGLT 2 Inhibitor):ti,ab,kw OR (Sodium-Glucose Transporter 2 Inhibitor):ti,ab,kw OR (Sodium Glucose Transporter 2 Inhibitor):ti,ab,kw OR (SGLT2 Inhibitor):ti,ab,kw OR (Inhibitor, SGLT2):ti,ab,kw OR (Gliflozins):ti,ab,kw OR (Gliflozin):ti,ab,kw OR (SGLT2 Inhibitors):ti,ab,kw OR (empagliflozin):ti,ab,kw OR (canagliflozin):ti,ab,kw OR (dapagliflozin ):ti,ab,kw OR (Ertugliflozin):ti,ab,kw OR (Ipragliflozin):ti,ab,kw OR (Luseogliflozin):ti,ab,kw OR (Tofogliflozin):ti,ab,kw OR (Cangrelor):ti,ab,kw |
| #4 | (non-steroid mineralocorticoid receptor antagonists):ti,ab,kw OR (nonsteroid mineralocorticoid receptor antagonists):ti,ab,kw OR (finerenone):ti,ab,kw OR (esaxerenone):ti,ab,kw OR (apararenone):ti,ab,kw |
| #5 | ("randomized clinical trial"):ti,ab,kw |
| #6 | #2 OR #3 OR #4 |
| #7 | #1 AND #5 AND #6 |

# 2.Table S1. Distribution of potential effect modifiers across included trials

| **Trial (year)** | **Intervention drug** | **N total** | **N intervention** | **N control** | **Age, years (I)** | **Age, years (C)** | **Male, % (I)** | **Male, % (C)** | **eGFR, mL/min/1.73m² (I)** | **eGFR, mL/min/1.73m² (C)** | **UACR, mg/g (I)** | **UACR, mg/g (C)** | **HbA1c, % (I)** | **HbA1c, % (C)** | **BMI, kg/m² (I)** | **BMI, kg/m² (C)** | **Diabetes duration, y (I)** | **Diabetes duration, y (C)** | **Follow-up** | **RASi use, % (I)** | **RASi use, % (C)** | **Overall RoB** |
| --- | --- | --- | --- | --- | --- | --- | --- | --- | --- | --- | --- | --- | --- | --- | --- | --- | --- | --- | --- | --- | --- | --- |
| FIDELIO-DKD,2020 | Finerenone | 5674 | 2833 | 2841 | 65.4 ± 8.9 | 65.7 ± 9.2 | 68.9 | 71.5 | 44.4 ± 12.5 | 44.3 ± 12.6 | 833 (441; 1628) | 867 (453; 1645) | 7.7 ± 1.3 | 7.7 ± 1.4 | 31.1 ± 6.0 | 31.1 ± 6.0 | 16.6 ± 8.8 | 16.6 ± 8.8 | 2.6 years | 99.9 | 99.9 | Low |
| FIGARO-DKD,2021 | Finerenone | 7352 | 3686 | 3666 | 64.1 ± 9.7 | 64.1 ± 10.0 | 68.6 | 70.3 | 67.6±21.7 | 68.0±21.7 | 302 (105; 749) | 315 (111; 731) | 7.7±1.4 | 7.7±1.4 | 31.5±6.0 | 31.4±5.9 | 14.5±8.6 | 14.4±8.4 | 3.4 years | 99.9 | 99.9 | Some concerns |
| LEADER,2016 | Liraglutide | 2158 | 1116 | 1042 | 67.3 ± 7.5 | 67.3 ± 7.5 | 61.9 | 60.6 | 45.5 ± 10.9 | 45.8 ± 10.8 | 47.3 (7.8; 236.8) | 51.8 (8.0; 305.9) | 8.7 ± 1.6 | 8.6 ± 1.5 | 32.6 ± 6.4 | 32.7 ± 6.6 | 15.4 ± 8.7 | 14.9 ± 8.5 | 26 weeks | 83.5 | 82.0 | Low |
| SUSTAIN-6,2016 | Semaglutide (s.c.) | 939 | 469 | 470 | N/A | N/A | N/A | N/A | N/A | N/A | N/A | N/A | N/A | N/A | N/A | N/A | N/A | N/A | 104 weeks | 83.6 | 83.4 | Low |
| EXSCEL,2017 | exenatide | 3177 | 1557 | 1620 | N/A | N/A | N/A | N/A | N/A | N/A | N/A | N/A | N/A | N/A | N/A | N/A | N/A | N/A | 3.2 years | N/A | N/A | Low |
| REWIND,2019 | Dulaglutide | 2199 | 1081 | 1118 | N/A | N/A | N/A | N/A | N/A | N/A | N/A | N/A | N/A | N/A | N/A | N/A | N/A | N/A | 5.4 years | 81 | 82 | Low |
| PIONEER 6,2019 | Oral semaglutide | 856 | 434 | 422 | N/A | N/A | N/A | N/A | N/A | N/A | N/A | N/A | N/A | N/A | N/A | N/A | N/A | N/A | 15.9 months | N/A | N/A | Low |
| FLOW,2024 | Semaglutide (s.c.) | 3533 | 1767 | 1766 | 66.6 ± 9.0 | 66.7 ± 9.0 | 70.6 | 68.9 | 46.9 ± 15.6 | 47.1 ± 14.7 | 582.3(Median) | 557.8(Median) | 7.8 ± 1.3 | 7.8 ± 1.3 | 31.9 ± 6.1 | 32.0 ± 6.5 | N/A | N/A | 3.4 years | 95.7 | 94.9 | Low |
| EMPA-REG RENAL,2014 | Empagliflozin | 374 | 187 | 187 | 64.6± 8.9 | 65.1± 8.2 | 57.2 | 56.7 | 45.4± 10.2 | 44.3± 10.3 | N/A | N/A | 8.02± 0.84 | 8.09± 0,80 | 30.2± 5.3 | 30.3± 5.3 | N/A | N/A | 52 weeks | 77.5 | 71.7 | Some concerns |
| Kohan,2014 | Dapagliflozin | 252 | 168 | 84 | 67± 8.3 | 67 ± 8.6 | 66.1 | 63.1 | 43.88± 10.1 | 45.84± 11.23 | N/A | N/A | 8.26± 1.01 | 8.53± 1.28 | N/A | N/A | 17.6± 9.6 | 15.7± 9.5 | 104 weeks | N/A | N/A | Some concerns |
| Yale, 2014* | Canagliflozin | 269 | 179 | 90 | 68.7± 8.2 | 68.2± 8.4 | 59.2 | 63.3 | 39.1 ± 6.9 | 40.1 ± 6.8 | 257.2 ± 644.6 | 255.1 ± 468.8 | 7.9±0.9 | 8.0±0.9 | 32.9±6.02 | 33.1±6.5 | 16.3±7.6 | 16.4±10.1 | 52 weeks | 88.3 | 85.6 | High |
| EMPA-REG OUTCOME,2015 | Empagliflozin | 1819 | 1212 | 607 | 67.1±7.6 | 67.1±8.2 | 67.3 | 68.9 | 48.4 ± 8.2 | 48.6 ± 7.8 | N/A | N/A | 8.07±0.86 | 8.03±0.85 | 31.0±5.5 | 30.9±5.4 | N/A | N/A | 3.1 years | 85.1 | 82.7 | Low |
| CANVAS,2017 | Canagliflozin | 2039 | 1110 | 929 | 67.6 ± 7.8 | 67.6 ± 7.6 | 59 | 57 | 49.2 ± 7.8 | 49.0 ± 8.3 | 21.5 (7.5; 132.7) | 21.7 (7.9; 105.5) | 8.3 ± 1.0 | 8.3 ± 0.9 | 32.1 ± 5.9 | 32.5 ± 6.2 | 16.1 ± 8.4 | 15.7± 8.2 | 188.2 weeks | 82 | 80 | Low |
| DERIVE,2018 | Dapagliflozin | 321 | 160 | 161 | 66 | 68 | 56.9 | 56.5 | 53.3 ± 8.7 | 53.6 ± 10.6 | 23.5 (2.7; 5852.0) | 29.0 (3.8,8474.0) | 8.33 ± 1.08 | 8.03 ± 1.08 | 32.6 ± 4.7 | 31.6 ± 5.0 | 14.3 ± 8.1 | 14.5 ± 8.3 | 24 weeks | 85.6 | 82 | Some concerns |
| DELIGHT，2019 | Dapagliflozin | 293 | 145 | 148 | 64.7 ± 8.6 | 64.7 ± 8.5 | 70 | 71 | 50.2 ± 13.0 | 47.7 ± 13.5 | 270.0 (69; 751) | 257.5 (80; 949) | 8.44 ± 1.0 | 8.57 ± 1.2 | 30.19 ± 5.3 | 30.34 ±5.6 | 17.55 ± 7.7 | 17.71 ± 9.5 | 24 weeks | 99 | 99 | Low |
| Allegretti,2019 | Ertugliflozin | 312 | 157 | 155 | 69.3 ± 8.36 | 69.9 ± 8.29 | 58.6 | 67.1 | 45.44 ± 8.6 | 44.78 ± 8.1 | N/A | N/A | 8.01 ± 0.786 | 7.95 ± 0.812 | 30.29 ± 5.988 | 30.1 ± 5.774 | 15.54 ± 9.918 | 16.28 ± 8.977 | 24 weeks | 72 | 75.5 | Low |
| CREDENCE,2019 | Canagliflozin | 4401 | 2202 | 2199 | 62.9 ± 9.2 | 63.2±9.2 | 65.4 | 66.7 | 56.3±18.2 | 56.0±18.3 | 923(459–1794) | 931(473–1868) | 8.3±1.3 | 8.3±1.3 | 31.4±6.2 | 31.3±6.2 | 15.5±8.7 | 16.0±8.6 | 2.62 years | 99.9 | 99.8 | Low |
| DECLARE-TIMI 58,2019 | Dapagliflozin | 1265 | 606 | 659 | N/A | N/A | N/A | N/A | N/A | N/A | N/A | N/A | N/A | N/A | N/A | N/A | N/A | N/A | 4.2 years | 81.3 | 81.3 | Low |
| VERTIS CV,2020 | Ertugliflozin | 1176 | 1178 | 598 | 68.3 ±7.6 | 68.0 ± 7.5 | 63.4 | 66.1 | 49.5 ± 7.5 | 49 ± 7.5 | N/A | N/A | 8.2 ±0.9 | 8.2 ±0.9 | 32.3 ± 5.5 | 32.6 ± 5.6 | 15.3 ± 8.7 | 16.4 ± 9.3 | 3.5 years | N/A | N/A | Low |
| DAPA-CKD,2020 | Dapagliflozin | 2906 | 1455 | 1451 | 64.1 ±9.8 | 64.7 ±9.5 | 66 | 68 | 44.0± 12.6 | 43.6± 12.6 | 1024.5 (472.5; 2111.0) | 1004.5 (493.3; 2017.0) | 7.8 ± 1.7 | 7.8 ± 1.6 | N/A | N/A | N/A | N/A | 2.4 years | 99 | 98 | Low |
| SCORED,2021 | Sotagliflozin | 10584 | 5292 | 5292 | N/A | N/A | 55.7 | 54.5 | N/A | N/A | 74 (18;486) | 75 (17;477) | N/A | N/A | N/A | N/A | N/A | N/A | 16.0 months | 87.5 | 86.9 | Low |

3.ROB 2.0 Risk Bias Assessment Chart：Detailed risk of bias in each study

## Domain 1: Risk of bias arising from the randomization process

| **Domain 1. Randomization process** | | | | | |  |
| --- | --- | --- | --- | --- | --- | --- |
| Study ID | 1.1 | 1.2 | 1.3 | 1 Algorithm results | 1Assessor's Judgement |  |
|  |  |  |  |  |  |  |
|  |  |  |  |  |  |  |
| FIDELIO-DKD,2020 | PY | Y | N | Low | Low |  |
| FIGARO-DKD,2021 | PY | Y | N | Low | Low |  |
| LEADER,2016 | Y | Y | N | Low | Low |  |
| SUSTAIN-6,2016 | Y | Y | N | Low | Low |  |
| EXSCEL,2017 | Y | Y | N | Low | Low |  |
| REWIND,2019 | Y | Y | N | Low | Low |  |
| PIONEER 6,2019 | Y | Y | N | Low | Low |  |
| FLOW,2024 | Y | Y | N | Low | Low |  |
| EMPA-REG RENAL,2014 | Y | Y | N | Low | Low |  |
| Kohan,2014 | PY | NI | N | Some concerns | Some concerns |  |
| Yale, 2014 | PY | NI | N | Some concerns | Some concerns |  |
| EMPA-REG OUTCOME,2015 | Y | Y | N | Low | Low |  |
| CANVAS,2017 | Y | Y | N | Low | Low |  |
| DERIVE,2018 | Y | Y | N | Low | Low |  |
| DELIGHT，2019 | Y | Y | N | Low | Low |  |
| Allegretti,2019 | Y | Y | N | Low | Low |  |
| CREDENCE,2019 | Y | Y | N | Low | Low |  |
| DECLARE-TIMI 58,2019 | Y | Y | N | Low | Low |  |
| VERTIS CV,2020 | Y | Y | N | Low | Low |  |
| DAPA-CKD,2020 | Y | Y | N | Low | Low |  |
| SCORED,2021 | Y | Y | N | Low | Low |  |

## Domain 2: Risk of bias due to deviations from the intended interventions

| **Domain 2. Deviations from intended interventions** | | | | | | | | | |
| --- | --- | --- | --- | --- | --- | --- | --- | --- | --- |
| Study ID | 2.1 | 2.2 | 2.3 | 2.4 | 2.5 | 2.6 | 2.7 | 2.0 Algorithm | 2.0 Assessor's |
|  |  |  |  |  |  |  |  | result | Judgement |
| FIDELIO-DKD,2020 | N | N | NA | NA | NA | PY | NA | Low | Low |
| FIGARO-DKD,2021 | N | N | NA | NA | NA | PN | PN | Some concerns | Some concerns |
| LEADER,2016 | N | N | NA | NA | NA | Y | NA | Low | Low |
| SUSTAIN-6,2016 | N | N | NA | NA | NA | Y | NA | Low | Low |
| EXSCEL,2017 | N | N | NA | NA | NA | Y | NA | Low | Low |
| REWIND,2019 | N | N | NA | NA | NA | Y | NA | Low | Low |
| PIONEER 6,2019 | N | N | NA | NA | NA | Y | NA | Low | Low |
| FLOW,2024 | N | N | NA | NA | NA | PY | NA | Low | Low |
| EMPA-REG RENAL,2014 | N | N | NA | NA | NA | PN | PN | Some concerns | Some concerns |
| Kohan,2014 | N | N | NA | NA | NA | PN | PN | Some concerns | Some concerns |
| Yale, 2014 | N | N | NA | NA | NA | PY | NA | Low | Low |
| EMPA-REG OUTCOME,2015 | N | N | NA | NA | NA | Y | NA | Low | Low |
| CANVAS,2017 | N | N | NA | NA | NA | Y | NA | Low | Low |
| DERIVE,2018 | N | N | NA | NA | NA | PN | N | Some concerns | Some concerns |
| DELIGHT，2019 | N | N | NA | NA | NA | Y | NA | Low | Low |
| Allegretti,2019 | N | N | NA | NA | NA | Y | NA | Low | Low |
| CREDENCE,2019 | N | N | NA | NA | NA | Y | NA | Low | Low |
| DECLARE-TIMI 58,2019 | N | N | NA | NA | NA | Y | NA | Low | Low |
| VERTIS CV,2020 | N | N | NA | NA | NA | Y | NA | Low | Low |
| DAPA-CKD,2020 | N | N | NA | NA | NA | Y | NA | Low | Low |
| SCORED,2021 | N | N | NA | NA | NA | Y | NA | Low | Low |

Domain 3: Risk of bias due to missing outcome data

| **Domain 3. Mising outcome data** | | | | | | |
| --- | --- | --- | --- | --- | --- | --- |
| Study ID | 3.1 | 3.2 | 3.3 | 3.4 | 3 | 3 |
|  |  |  |  |  | Algorithm | Assessor's judgement |
|  |  |  |  |  | result |  |
| FIDELIO-DKD,2020 | Y | NA | NA | NA | Low | Low |
| FIGARO-DKD,2021 | Y | NA | NA | NA | Low | Low |
| LEADER,2016 | Y | NA | NA | NA | Low | Low |
| SUSTAIN-6,2016 | Y | NA | NA | NA | Low | Low |
| EXSCEL,2017 | Y | NA | NA | NA | Low | Low |
| REWIND,2019 | Y | NA | NA | NA | Low | Low |
| PIONEER 6,2019 | Y | NA | NA | NA | Low | Low |
| FLOW,2024 | Y | NA | NA | NA | Low | Low |
| EMPA-REG RENAL,2014 | PN | PY | NA | NA | Low | Low |
| Kohan,2014 | N | PY | NA | NA | Low | Low |
| Yale, 2014 | N | N | PY | PY | High | High |
| EMPA-REG OUTCOME,2015 | Y | NA | NA | NA | Low | Low |
| CANVAS,2017 | Y | NA | NA | NA | Low | Low |
| DERIVE,2018 | PN | Y | NA | NA | Low | Low |
| DELIGHT，2019 | Y | NA | NA | NA | Low | Low |
| Allegretti,2019 | Y | NA | NA | NA | Low | Low |
| CREDENCE,2019 | Y | NA | NA | NA | Low | Low |
| DECLARE-TIMI 58,2019 | Y | NA | NA | NA | Low | Low |
| VERTIS CV,2020 | Y | NA | NA | NA | Low | Low |
| DAPA-CKD,2020 | Y | NA | NA | NA | Low | Low |
| SCORED,2021 | Y | NA | NA | NA | Low | Low |

Domain 4: Risk of bias in measurement of the outcome

| **Domain 4. Measurement of the outcome** | | | | | | | |
| --- | --- | --- | --- | --- | --- | --- | --- |
| Study ID | 4.1 | 4.2 | 4.3 | 4.4 | 4.5 | 4 | 4 |
|  |  |  |  |  |  | Algorithm result | Assessor's Judgement |
| FIDELIO-DKD,2020 | N | N | N | NA | NA | Low | Low |
| FIGARO-DKD,2021 | N | N | N | NA | NA | Low | Low |
| LEADER,2016 | N | N | N | NA | NA | Low | Low |
| SUSTAIN-6,2016 | N | N | N | NA | NA | Low | Low |
| EXSCEL,2017 | N | N | N | NA | NA | Low | Low |
| REWIND,2019 | N | N | N | NA | NA | Low | Low |
| PIONEER 6,2019 | N | N | N | NA | NA | Low | Low |
| FLOW,2024 | N | N | N | NA | NA | Low | Low |
| EMPA-REG RENAL,2014 | N | N | N | NA | NA | Low | Low |
| Kohan,2014 | N | N | N | NA | NA | Low | Low |
| Yale, 2014 | N | N | N | NA | NA | Low | Low |
| EMPA-REG OUTCOME,2015 | N | N | N | NA | NA | Low | Low |
| CANVAS,2017 | N | N | N | NA | NA | Low | Low |
| DERIVE,2018 | N | N | N | NA | NA | Low | Low |
| DELIGHT，2019 | N | N | N | NA | NA | Low | Low |
| Allegretti,2019 | N | N | N | NA | NA | Low | Low |
| CREDENCE,2019 | N | N | N | NA | NA | Low | Low |
| DECLARE-TIMI 58,2019 | N | N | N | NA | NA | Low | Low |
| VERTIS CV,2020 | N | N | N | NA | NA | Low | Low |
| DAPA-CKD,2020 | N | N | N | NA | NA | Low | Low |
| SCORED,2021 | PN | N | N | NA | NA | Low | Low |

Domain 5: Risk of bias in selection of the reported result

| **Domain 5. Selection of the reported result** | | | | | |
| --- | --- | --- | --- | --- | --- |
| Study ID | 5.1 | 5.2 | 5.3 | 5 | 5 |
|  |  |  |  | Algorithm result | Assessor's Judgement |
| FIDELIO-DKD,2020 | Y | N | N | Low | Low |
| FIGARO-DKD,2021 | Y | N | N | Low | Low |
| LEADER,2016 | Y | N | N | Low | Low |
| SUSTAIN-6,2016 | Y | N | N | Low | Low |
| EXSCEL,2017 | Y | N | N | Low | Low |
| REWIND,2019 | Y | N | N | Low | Low |
| PIONEER 6,2019 | Y | N | N | Low | Low |
| FLOW,2024 | Y | N | N | Low | Low |
| EMPA-REG RENAL,2014 | NI | PN | N | Some concerns | Some concerns |
| Kohan,2014 | Y | N | N | Low | Low |
| Yale, 2014 | Y | N | N | Low | Low |
| EMPA-REG OUTCOME,2015 | Y | N | N | Low | Low |
| CANVAS,2017 | Y | N | N | Low | Low |
| DERIVE,2018 | Y | N | N | Low | Low |
| DELIGHT，2019 | Y | N | N | Low | Low |
| Allegretti,2019 | Y | N | N | Low | Low |
| CREDENCE,2019 | Y | N | N | Low | Low |
| DECLARE-TIMI 58,2019 | Y | N | N | Low | Low |
| VERTIS CV,2020 | Y | N | N | Low | Low |
| DAPA-CKD,2020 | Y | N | N | Low | Low |
| SCORED,2021 | PY | N | PN | Low | Low |

Overall risk of bias

| **Overall Bias** | | |
| --- | --- | --- |
| Study ID | Algorithm's | Assessor's |
|  | overall Judgement | overall Judgement |
| FIDELIO-DKD,2020 | Low | Low |
| FIGARO-DKD,2021 | Some concerns | Some concerns |
| LEADER,2016 | Low | Low |
| SUSTAIN-6,2016 | Low | Low |
| EXSCEL,2017 | Low | Low |
| REWIND,2019 | Low | Low |
| PIONEER 6,2019 | Low | Low |
| FLOW,2024 | Low | Low |
| EMPA-REG RENAL,2014 | Some concerns | Some concerns |
| Kohan,2014 | Some concerns | Some concerns |
| Yale, 2014 | High | High |
| EMPA-REG OUTCOME,2015 | Low | Low |
| CANVAS,2017 | Low | Low |
| DERIVE,2018 | Some concerns | Some concerns |
| DELIGHT，2019 | Low | Low |
| Allegretti,2019 | Low | Low |
| CREDENCE,2019 | Low | Low |
| DECLARE-TIMI 58,2019 | Low | Low |
| VERTIS CV,2020 | Low | Low |
| DAPA-CKD,2020 | Low | Low |
| SCORED,2021 | Low | Low |

4 Network plot

4.1 CV death

Fig. S1 Network plot for CV death

4.2 All-cause death

Fig. S2 Network plot for All-cause death

4.3 HHF

Fig.S3 Network plot for HHF

**5. Treatment rankings and relative-effect estimates**

**5.1 Table S2. SUCRA values for the primary outcomes**

| **Treatment** | **MACE** | **Major kidney** **composite outcome** | **HHF** | **All-cause** **mortality** | **Cardiovascular** **death** |
| --- | --- | --- | --- | --- | --- |
| Placebo | 1.8 | 0.2 | 0.7 | 4.1 | 3.5 |
| GLP-1RAs | 72.1 | 48.1 | 60.9 | 78.2 | 98.7 |
| SGLT2is | 64.8 | 99.8 | 94.2 | 63.2 | 44.2 |
| nsMRAs | 61.3 | 51.9 | 44.2 | 54.5 | 53.5 |

**Note: Values are expressed as percentages. SUCRA, surface under the cumulative ranking curve; MACE, major adverse cardiovascular events; HHF, hospitalization for heart failure. Higher SUCRA values indicate higher relative rankings.**

**5.2 Table S3. Combined league table of relative effects for the five main outcomes**

| **A. MACE** | | | |
| --- | --- | --- | --- |
| **GLP-1RAs** | 1.02 (0.84–1.24) | 1.03 (0.82–1.29) | 1.17 (1.02–1.33) |
| 0.98 (0.81–1.19) | **SGLT2is** | 1.01 (0.82–1.25) | 1.15 (1.03–1.29) |
| 0.97 (0.77–1.22) | 0.99 (0.80–1.22) | **nsMRAs** | 1.14 (0.98–1.33) |
| 0.83 (0.72–0.97) | 0.85 (0.75–0.96) | 0.86 (0.72–1.02) | **Placebo** |
| **B. Major kidney composite outcome** | | | |
| **GLP-1RAs** | 0.77 (0.63–0.94) | 0.99 (0.83–1.17) | 1.18 (1.03–1.36) |
| 1.30 (1.07–1.59) | **SGLT2is** | 1.29 (1.08–1.53) | 1.54 (1.34–1.77) |
| 1.01 (0.85–1.20) | 0.78 (0.66–0.92) | **nsMRAs** | 1.20 (1.09–1.32) |
| 0.84 (0.73–0.97) | 0.65 (0.56–0.75) | 0.83 (0.75–0.92) | **Placebo** |
| **C. Hospitalization for heart failure** | | | |
| **GLP-1RAs** | 0.84 (0.59–1.19) | 1.09 (0.77–1.54) | 1.40 (1.03–1.90) |
| 1.19 (0.84–1.70) | **SGLT2is** | 1.30 (1.02–1.67) | 1.67 (1.39–2.01) |
| 0.92 (0.65–1.30) | 0.77 (0.60–0.98) | **nsMRAs** | 1.29 (1.09–1.52) |
| 0.71 (0.53–0.97) | 0.60 (0.50–0.72) | 0.78 (0.66–0.92) | **Placebo** |
| **D. All-cause mortality** | | | |
| **GLP-1RAs** | 1.04 (0.84–1.29) | 1.06 (0.85–1.34) | 1.20 (1.03–1.40) |
| 0.96 (0.78–1.19) | **SGLT2is** | 1.02 (0.82–1.28) | 1.15 (0.99–1.34) |
| 0.94 (0.75–1.18) | 0.98 (0.78–1.22) | **nsMRAs** | 1.13 (0.95–1.33) |
| 0.83 (0.72–0.97) | 0.87 (0.75–1.01) | 0.89 (0.75–1.05) | **Placebo** |
| **E. Cardiovascular death** | | | |
| **GLP-1RAs** | 1.31 (1.04–1.65) | 1.27 (1.00–1.62) | 1.45 (1.20–1.75) |
| 0.76 (0.61–0.96) | **SGLT2is** | 0.97 (0.79–1.19) | 1.11 (0.97–1.26) |
| 0.79 (0.62–1.00) | 1.03 (0.84–1.26) | **nsMRAs** | 1.14 (0.98–1.33) |
| 0.69 (0.57–0.83) | 0.90 (0.79–1.03) | 0.88 (0.75–1.02) | **Placebo** |

***Note:* Values are RRs with 95% CIs. Each cell presents the effect of the column-defining treatment relative to the row-defining treatment; an RR below 1.0 favors the column-defining treatment. Estimates on opposite sides of the diagonal are reciprocal orientations. Comparisons between active drug classes were based exclusively on indirect evidence. MACE, major adverse cardiovascular events; HHF, hospitalization for heart failure; GLP-1RAs, glucagon-like peptide-1 receptor agonists; SGLT2is, sodium–glucose cotransporter-2 inhibitors; nsMRAs, nonsteroidal mineralocorticoid receptor antagonists.**

**5.3 Table S4. Contribution of individual trials to the five main outcome networks**

| **Study ID** | **Trial** | **Year** | **Drug class** | **MACE** | **Major kidney composite outcome** | **HHF** | **All-cause mortality** | **Cardiovascular death** |
| --- | --- | --- | --- | --- | --- | --- | --- | --- |
| 1 | FIDELIO-DKD | 2020 | nsMRA | ✓ | ✓ | ✓ | ✓ | ✓ |
| 2 | FIGARO-DKD | 2021 | nsMRA | ✓ | ✓ | ✓ | ✓ | ✓ |
| 3 | LEADER | 2016 | GLP-1RA | ✓ | — | ✓ | ✓ | ✓ |
| 4 | SUSTAIN-6 | 2016 | GLP-1RA | ✓ | — | — | — | — |
| 5 | EXSCEL | 2017 | GLP-1RA | ✓ | — | — | ✓ | — |
| 6 | REWIND | 2019 | GLP-1RA | — | ✓ | — | — | — |
| 7 | PIONEER 6 | 2019 | GLP-1RA | ✓ | — | — | — | — |
| 8 | FLOW | 2024 | GLP-1RA | ✓ | ✓ | — | ✓ | ✓ |
| 9 | EMPA-REG RENAL | 2014 | SGLT2i | — | — | — | — | — |
| 10 | Kohan | 2014 | SGLT2i | — | — | — | — | — |
| 11 | Yale | 2014 | SGLT2i | — | — | — | — | — |
| 12 | EMPA-REG OUTCOME | 2015 | SGLT2i | ✓ | — | — | — | ✓ |
| 13 | CANVAS | 2017 | SGLT2i | ✓ | ✓ | ✓ | — | ✓ |
| 14 | DERIVE | 2018 | SGLT2i | — | — | — | — | — |
| 15 | DELIGHT | 2019 | SGLT2i | — | — | — | — | — |
| 16 | Allegretti | 2019 | SGLT2i | — | — | — | — | — |
| 17 | CREDENCE | 2019 | SGLT2i | ✓ | ✓ | ✓ | ✓ | — |
| 18 | DECLARE-TIMI 58 | 2019 | SGLT2i | ✓ | ✓ | ✓ | ✓ | ✓ |
| 19 | VERTIS CV | 2020 | SGLT2i | ✓ | — | ✓ | — | ✓ |
| 20 | DAPA-CKD | 2020 | SGLT2i | — | ✓ | — | ✓ | ✓ |
| 21 | SCORED | 2021 | SGLT2i | ✓ | ✓ | — | ✓ | ✓ |
|  | **Number of contributing RCTs** |  |  | **13** | **9** | **7** | **9** | **10** |

**Note: ✓ indicates that the trial contributed eligible data to the corresponding network meta-analysis; — indicates that no eligible data were included for that outcome. For some cardiovascular outcome trials, CKD-specific outcome data were obtained from prespecified subgroup or secondary analyses; these publications were used as data sources and were not counted as separate RCTs. MACE, major adverse cardiovascular events; HHF, hospitalization for heart failure; CKD, chronic kidney disease; RCT, randomized controlled trial.**

***6. CINeMA Assessment of Evidence Certainty***

To evaluate the certainty of evidence for the five prespecified clinical hard endpoints in the network meta-analysis, the CINeMA (Confidence in Network Meta-Analysis) framework was applied. Metabolic outcomes were considered exploratory and were not included in the CINeMA assessment. Following CINeMA guidelines, each comparison was assessed across six domains: within-study bias, reporting bias, indirectness, imprecision, heterogeneity, and incoherence.Similar to the GRADE approach, the certainty of evidence was initially assumed to be “high” and downgraded based on concerns in each domain.

The specific criteria for each domain were as follows: Within-study bias: Based on the overall RoB 2.0 judgments of included studies. If more than 30% of studies contributing direct evidence were rated as having unclear or high risk of bias, the certainty was downgraded. Reporting bias: When ≥10 studies were included for an outcome, funnel plots (and comparison-adjusted funnel plots when necessary) were used to assess small-study effects/publication bias. When <10 studies were included, no formal assessment was conducted due to limited statistical power, and the domain was rated as “some concerns.” Indirectness: Assessed whether differences in population, interventions, outcomes, or study settings reduced applicability to the target clinical question. If substantial indirectness was present (e.g., differences in outcome definitions), the certainty was downgraded accordingly. Imprecision: A clinical decision threshold of 0.80–1.25 was applied. If the 95% CI lay entirely within this range, no clinically important difference was assumed. If the CI crossed this boundary, it was rated as “some concerns.” If the CI crossed the null line and included both clinically important benefit and potential harm (i.e., could lead to opposing clinical decisions), it was rated as “major concerns.” Heterogeneity: Assessed using CINeMA’s semi-automated approach, incorporating the network heterogeneity variance (τ²) and corresponding prediction intervals, as well as measures such as I². If heterogeneity was substantial (e.g., I² >50% or prediction intervals included clinically important differences), the certainty was downgraded. Incoherence (inconsistency): Evaluated the agreement between direct and indirect evidence. As the network lacked closed loops, formal inconsistency testing was not feasible; therefore, this domain was rated as “some concerns” for all comparisons.

Overall, the quality of evidence was relatively low(Fig S4-S8). Only some comparisons for cardiovascular death reached moderate certainty, while the majority of comparisons for other outcomes were rated as low or very low certainty. The primary reason for downgrading was the lack of direct head-to-head randomized controlled trials among the three target drug classes. All between-class comparisons relied on indirect evidence using placebo as a common comparator, resulting in networks without closed loops. Consequently, indirectness emerged as a consistent and dominant factor contributing to evidence downgrading across all outcomes. In addition, transitivity assessment revealed differences in key effect modifiers—such as proteinuria burden and follow-up duration—across studies, further increasing the risk of indirectness. For MACE and major kidney composite outcomes, evidence quality was additionally affected by inconsistencies in outcome definitions across studies. For example, some studies included hospitalization for heart failure in the definition of MACE, while renal composite outcomes varied in terms of eGFR decline thresholds and inclusion criteria for hard endpoints, introducing additional heterogeneity. For hospitalization for HHF, although comparisons between individual drugs and placebo were rated as low certainty, indirect comparisons between active agents were influenced by uneven evidence distribution. In particular, comparisons between GLP-1RAs and nsMRAs were associated with serious concerns regarding imprecision, representing a key limitation for this outcome. All-cause mortality had the lowest certainty among all outcomes, due to the combined impact of within-study bias, imprecision, and heterogeneity. In contrast, cardiovascular death was the only outcome with moderate certainty in some comparisons, owing to low between-study heterogeneity and highly consistent outcome definitions, making it the most robust endpoint in this analysis. The stringent multidimensional downgrading criteria of the CINeMA framework also highlight the inherent limitations of the current evidence base, indicating that most outcomes have not yet reached a high level of certainty for definitive clinical conclusions.

Fig S4.MACE CINeMa Evaluation Result

Fig S5. major kidney disease events CINeMa Evaluation Result

Fig S6.HHF CINeMa Evaluation Result

Fig S7.ACD CINeMa Evaluation Result

Fig S8.CVD CINeMa Evaluation Result

**7 *Exploratory Metabolic Outcomes***

Exploratory analyses indicated that the magnitude of improvement in surrogate metabolic markers—such as HbA1c, body weight (or BMI), blood pressure, and UACR—differed among the drug classes, providing complementary evidence for overall benefit assessment and potential mechanistic interpretation. For body weight change, the random-effects network meta-analysis (Fig. S9) showed that, compared with placebo, both GLP-1RAs (MD = −3.53 kg, 95% CI [−11.02 to 3.97]) and SGLT2is (MD = −1.31 kg, 95% CI [−1.64 to −0.97]) were associated with weight reduction; however, only the effect of SGLT2is reached statistical significance. Subgroup analysis revealed substantial heterogeneity within the GLP-1RA group (I² = 89.6%, P = 0.002), whereas heterogeneity within the SGLT2i group was low to moderate (I² = 29.1%, P = 0.206). The random-effects model demonstrated a significant subgroup difference between the two drug classes in their effects on body weight (P = 0.0002). These findings suggest that SGLT2is produce a consistent and statistically significant reduction in body weight, whereas GLP-1RAs may have a greater magnitude of weight loss, but the robustness of this effect is influenced by variability across included studies.

**Fig.S9 body weight change forest plot**

For HbA1c change, the random-effects meta-analysis (Fig. S10) showed that, compared with placebo, SGLT2is significantly reduced HbA1c (MD = −0.42, 95% CI [−0.47 to −0.38]), with moderate between-study heterogeneity (I² = 40.1%, P = 0.111). GLP-1RAs also demonstrated a reduction trend; however, the effect estimates from two key studies (LE ADER, 2016; FLOW, 2024) were −0.40 (95% CI [−0.53 to −0.27]) and −0.81 (95% CI [−0.90 to −0.72]), respectively, with substantial within-group heterogeneity (I² = 96.2%, P < 0.001). Subgroup analysis indicated a significant difference between the two drug classes in their effects on HbA1c (P < 0.0001). These results suggest that SGLT2is provide a consistent and statistically significant reduction in HbA1c, whereas the effect estimates for GLP-1RAs are more variable and influenced by differences across included studies.

**Fig.S10 HbAlc change forest plot**

For systolic blood pressure (SBP) change, the random-effects meta-analysis (Fig. S11) showed that, compared with placebo, SGLT2is significantly reduced SBP (MD = −3.48 mmHg, 95% CI [−4.13 to −2.83]), with no heterogeneity across studies (I² = 0%, P = 0.635). GLP-1RAs also showed a reduction trend; however, the effect estimates from two key studies (LEADER, 2016; FLOW, 2024) were −0.15 (95% CI [−1.72 to 1.42]) and −2.23 (95% CI [−3.32 to −1.14]), respectively, with moderate heterogeneity within the group (I² = 78.3%, P = 0.032). Subgroup analysis indicated no statistically significant difference between the two drug classes in their effects on SBP (P = 0.128). These findings suggest that SGLT2is provide a consistent and statistically significant reduction in SBP, whereas the effect estimates for GLP-1RAs are more variable and influenced by differences across included studies.

Fig.S11 SBP change forest plot

***8 Sensitivity Analyses***

Given the differences in study design and patient characteristics between CKD subgroup data derived from cardiovascular outcome trials (CVOTs) and CKD-specific trials, as well as variation in kidney-outcome definitions, post hoc sensitivity analyses focusing on MACE and the major kidney composite outcome were conducted to assess the robustness of the principal cardiorenal findings.

***8.1 MACE***

To assess whether differences in trial design influenced the comparative estimates for MACE, studies were stratified into CKD-specific trials and CVOT-derived CKD subgroup data, and the network meta-analysis was repeated within each stratum. Among CKD-specific trials, GLP-1RAs (RR 0.83, 95% CI 0.70–0.99), SGLT2is (RR 0.79, 95% CI 0.71–0.88), and nsMRAs (RR 0.88, 95% CI 0.80–0.96) were each associated with a lower MACE risk than placebo. However, none of the between-class comparisons was statistically significant. Between-study heterogeneity was low (τ² ≈ 0; I² = 0.0%; P = 0.944; Fig. S12).

**Fig.S12 Sensitivity analysis of MACE restricted to CKD-specific trials**

In the CVOT-derived CKD subgroup analysis, only GLP-1RAs and SGLT2is were represented because no eligible nsMRA CVOT was available. Neither GLP-1RAs (RR 0.86, 95% CI 0.72–1.03) nor SGLT2is (RR 0.93, 95% CI 0.79–1.10) differed significantly from placebo, and no significant difference was observed between SGLT2is and GLP-1RAs (RR 1.08, 95% CI 0.85–1.38). Moderate heterogeneity was observed (τ² = 0.0143; I² = 49.7%; P = 0.063; Fig. S13).

**Fig.S13 Sensitivity analysis of MACE using CKD subgroup data derived from cardiovascular outcome trials.**

Despite differences in the placebo-controlled estimates between the 2 strata, neither analysis identified a statistically significant difference between the available active drug classes, consistent with the overall analysis. Thus, stratification by trial design did not materially alter the available between-class findings for MACE.Nevertheless, the results should be interpreted cautiously because the stratified networks were smaller and the CVOT-derived subgroup lacked an nsMRA node.

***8.2 Major Kidney Composite Outcome***

***8.2.1 Sensitivity analyses according to trial design***

To assess whether differences in trial design influenced the comparative estimates for the major kidney composite outcome, studies were stratified into CKD-specific trials and CVOT-derived CKD subgroup data, and the network meta-analysis was repeated within each stratum.

In the CKD-specific trials, GLP-1RAs (RR 0.84, 95% CI 0.71–0.99), SGLT2is (RR 0.65, 95% CI 0.57–0.75), and nsMRAs (RR 0.86, 95% CI 0.79–0.93) were each associated with a lower risk of the major kidney composite outcome than placebo. The network estimates favored SGLT2is over GLP-1RAs (RR 0.78, 95% CI 0.62–0.97) and nsMRAs (nsMRAs versus SGLT2is: RR 1.32, 95% CI 1.12–1.55), whereas no significant difference was observed between nsMRAs and GLP-1RAs (RR 1.02, 95% CI 0.85–1.23). Between-study heterogeneity was low (τ² ≈ 0; I² = 0.0%; P = 0.741; Fig. S14).

**Fig. S14. Sensitivity analysis of the major kidney composite outcome restricted to CKD-specific trials.**

In the CVOT-derived CKD subgroup analysis, only GLP-1RAs and SGLT2is were represented because no eligible nsMRA data were available. Neither GLP-1RAs (RR 0.90, 95% CI 0.64–1.26) nor SGLT2is (RR 0.76, 95% CI 0.51–1.13) showed a statistically significant difference from placebo, and the indirect comparison between SGLT2is and GLP-1RAs was also not significant (RR 0.84, 95% CI 0.50–1.42). Between-study heterogeneity was low to moderate (τ² = 0.0229; I² = 28.1%; P = 0.238; Fig. S15).

**Fig. S15. Sensitivity analysis of the major kidney composite outcome using CKD subgroup data derived from cardiovascular outcome trials.**

The direction of the available estimates was broadly consistent across the 2 trial-design strata and with the overall analysis, with SGLT2is showing numerically greater renal protection than GLP-1RAs. The CVOT-derived estimate for SGLT2is versus GLP-1RAs was directionally similar to that from CKD-specific trials but was substantially less precise, with a wider confidence interval.Thus, the principal pattern of the kidney findings remained generally stable, although the CVOT-derived estimates were less precise and the absence of an nsMRA node precluded a complete 3-class comparison.

***8.2.2 Sensitivity Analyses According to Major Kidney Composite Outcome Definitions***

The definitions of the major kidney composite outcome varied across the included trials, particularly with respect to the threshold for eGFR decline and the inclusion of albuminuria-related components. During data extraction, we retained the trial-defined major kidney composite outcome and recorded its individual components. Event counts were not recalculated or converted across different endpoint definitions. To assess the robustness of the findings to this variation, we performed 2 progressively restrictive post hoc sensitivity analyses. Trial-specific definitions of the major kidney composite outcome and their eligibility for the endpoint-definition sensitivity analyses are summarized in Supplementary Table S5.

First, we excluded the REWIND trial because its renal composite included new-onset macroalbuminuria and a sustained ≥30% decline in eGFR, representing a broader endpoint than those used in the other trials. After its exclusion, the estimates remained broadly consistent with the main analysis. The RRs versus placebo were 0.84 (95% CI 0.71–0.99) for GLP-1RAs, 0.67 (95% CI 0.59–0.76) for SGLT2is, and 0.86 (95% CI 0.79–0.93) for nsMRAs. Among the active treatments, the RRs were 0.80 (95% CI 0.64–0.99) for SGLT2is versus GLP-1RAs, 1.02 (95% CI 0.85–1.23) for nsMRAs versus GLP-1RAs, and 1.28 (95% CI 1.10–1.50) for nsMRAs versus SGLT2is. Between-study heterogeneity was negligible (τ² ≈ 0; I² = 0.0%; P = 0.631), indicating that the broader renal composite used in REWIND did not materially influence the principal findings (Fig. S16).

**Fig. S16. Sensitivity analysis of the major kidney composite outcome after exclusion of the REWIND trial.**

Second, we restricted the analysis to trials in which the renal-function deterioration component was defined as a sustained ≥50% decline in eGFR or doubling of serum creatinine and the composite additionally included kidney failure, end-stage kidney disease, long-term dialysis or kidney replacement therapy, or renal death. In this more stringent analysis, the RRs versus placebo were 0.84 (95% CI 0.71–0.99) for GLP-1RAs and 0.67 (95% CI 0.59–0.77) for SGLT2is. The RR for SGLT2is versus GLP-1RAs was 0.80 (95% CI 0.65–1.00), with the upper confidence limit reaching the null value. An estimate for nsMRAs could not be generated because the eligible nsMRA trials defined kidney-function deterioration using a sustained ≥40% decline in eGFR and were therefore excluded from this stringent analysis. The estimated between-study heterogeneity was negligible (τ² ≈ 0; I² = 0.6%; heterogeneity-test P = 0.389; Fig. S17).

**Fig. S17. Sensitivity analysis of the major kidney composite outcome restricted to trials using stringent kidney-function endpoint definitions.**

Overall, progressively restricting the kidney outcome definition produced only small changes in the available effect estimates, and their directions remained consistent with the main analysis. However, the reduced precision of the comparison between SGLT2is and GLP-1RAs and the loss of the nsMRA node in the most stringent analysis indicate that these findings should be interpreted in the context of the smaller and less complete network.

**Table S5. Trial-specific definitions of the major kidney composite outcome and inclusion in endpoint-definition sensitivity analyses**

| **Trial, year** | **Drug class** | **Source of included outcome data** | **Trial-defined major kidney composite outcome** | **Analysis excluding REWIND** | **Stringent-definition analysis** |
| --- | --- | --- | --- | --- | --- |
| FIDELIO-DKD, 2020 | nsMRA | CKD-specific trial | Kidney failure, sustained ≥40% decline in eGFR, or renal death | Included | Excluded |
| FIGARO-DKD, 2021 | nsMRA | CKD-specific trial | Kidney failure, sustained ≥40% decline in eGFR, or renal death | Included | Excluded |
| REWIND, 2019 | GLP-1RA | CKD subgroup derived from a CVOT | New-onset macroalbuminuria, sustained ≥30% decline in eGFR, or kidney replacement therapy | Excluded | Excluded |
| FLOW, 2024 | GLP-1RA | CKD-specific trial | Kidney failure (persistent eGFR <15 mL/min/1.73 m²), sustained ≥50% decline in eGFR, kidney replacement therapy, or renal death | Included | Included |
| CANVAS, 2017 | SGLT2i | CKD subgroup derived from a CVOT | End-stage kidney disease, doubling of serum creatinine, or renal death | Included | Included |
| CREDENCE, 2019 | SGLT2i | CKD-specific trial | End-stage kidney disease, doubling of serum creatinine, or renal death | Included | Included |
| DECLARE-TIMI 58, 2019 | SGLT2i | CKD subgroup derived from a CVOT | End-stage kidney disease, sustained ≥40% decline in eGFR, or renal death | Included | Excluded |
| DAPA-CKD, 2020 | SGLT2i | CKD-specific trial (eligible T2D subgroup) | End-stage kidney disease, sustained ≥50% decline in eGFR, or renal death | Included | Included |
| SCORED, 2021 | SGLT2i | CKD-specific trial | Persistent eGFR <15 mL/min/1.73 m², sustained ≥50% decline in eGFR, long-term dialysis, or kidney replacement therapy | Included | Included |

Notes: Definitions and component event counts were retained as originally reported; events were not reconstructed or converted across definitions. The stringent-definition analysis was restricted at the trial level to composites defining kidney-function deterioration as a sustained ≥50% decline in eGFR or doubling of serum creatinine and also including kidney failure or end-stage kidney disease, long-term dialysis or kidney replacement therapy, or renal death. CKD, chronic kidney disease; CVOT, cardiovascular outcome trial; eGFR, estimated glomerular filtration rate; GLP-1RA, glucagon-like peptide-1 receptor agonist; nsMRA, nonsteroidal mineralocorticoid receptor antagonist; SGLT2i, sodium-glucose cotransporter-2 inhibitor; T2D, type 2 diabetes.

***9 Additional Analyses: Drug-Level Comparisons***

To explore potential within-class variation, we separated the class-level treatment nodes into individual agents and repeated the network meta-analyses at the drug level. Complete pairwise relative-effect estimates are presented in Tables S6–S10. These analyses were exploratory because most comparisons between active agents were informed indirectly through placebo and were frequently imprecise.

For MACE, several nominal pairwise differences were observed, although most 95% CIs crossed the line of no effect and the estimates did not show a consistent pattern of differences among agents within the same drug class (Table S6).

For the major kidney composite outcome, dapagliflozin, canagliflozin, subcutaneous semaglutide and finerenone were associated with lower risks than placebo. Among active agents, the indirect estimates favoured dapagliflozin over subcutaneous semaglutide (RR 0.70, 95% CI 0.51–0.94), finerenone (RR 0.68, 95% CI 0.53–0.87) and dulaglutide (RR 0.65, 95% CI 0.47–0.88). The remaining active-agent comparisons did not provide evidence of statistically significant differences (Table S7).

For HHF, ertugliflozin, canagliflozin, liraglutide and finerenone were associated with lower risks than placebo, whereas the estimate for dapagliflozin was imprecise and crossed the line of no effect. Among active agents, the only statistically significant indirect estimate favoured ertugliflozin over finerenone (RR 0.63, 95% CI 0.40–0.99); the remaining comparisons did not show statistically significant differences (Table S8).

For all-cause mortality, liraglutide, subcutaneous semaglutide and dapagliflozin were associated with lower risks than placebo. The indirect estimates also favoured liraglutide over sotagliflozin (RR 0.72, 95% CI 0.53–0.97) and exenatide (RR 0.71, 95% CI 0.51–0.97). No statistically significant differences were identified in the other active-agent comparisons (Table S9).

For cardiovascular death, liraglutide and subcutaneous semaglutide were associated with lower risks than placebo. The indirect estimates also favoured liraglutide (RR 0.57, 95% CI 0.36–0.90) and subcutaneous semaglutide (RR 0.61, 95% CI 0.40–0.93) over ertugliflozin. The remaining active-agent comparisons did not show statistically significant differences (Table S10).

Overall, the drug-level analyses suggested outcome-specific differences rather than a consistent treatment hierarchy across outcomes. Given the sparse networks, the absence of head-to-head comparisons between active agents and the uncertainty surrounding many estimates, these exploratory findings should be interpreted cautiously.

**Table S6. Drug-level league table for MACE**

| MACE | | | | | | | | | | |
| --- | --- | --- | --- | --- | --- | --- | --- | --- | --- | --- |
| Liraglutide | Sotagliflozin | Semaglutide oral | Canagliflozin | Semaglutide SC | Finerenone | Dapagliflozin | Empagliflozin | Placebo | Exenatide | Ertugliflozin |
| Liraglutide | 0.93 (0.74–1.16) | 0.93 (0.51–1.69) | 0.87 (0.69–1.09) | 0.86 (0.68–1.09) | 0.82 (0.67–1.00) | 0.81 (0.59–1.12) | 0.81 (0.61–1.08) | 0.72 (0.60–0.86) | 0.70 (0.55–0.88) | 0.66 (0.50–0.88) |
| 1.08 (0.86–1.35) | Sotagliflozin | 1.00 (0.55–1.80) | 0.94 (0.77–1.14) | 0.93 (0.75–1.14) | 0.88 (0.75–1.04) | 0.87 (0.65–1.18) | 0.87 (0.67–1.13) | 0.78 (0.68–0.89) | 0.75 (0.61–0.92) | 0.72 (0.56–0.92) |
| 1.08 (0.59–1.97) | 1.00 (0.56–1.81) | Semaglutide oral | 0.94 (0.52–1.69) | 0.93 (0.51–1.68) | 0.89 (0.50–1.58) | 0.88 (0.47–1.64) | 0.87 (0.47–1.62) | 0.78 (0.44–1.38) | 0.75 (0.42–1.36) | 0.72 (0.39–1.32) |
| 1.15 (0.92–1.44) | 1.07 (0.88–1.29) | 1.06 (0.59–1.92) | Canagliflozin | 0.99 (0.81–1.22) | 0.94 (0.80–1.11) | 0.93 (0.69–1.25) | 0.93 (0.71–1.21) | 0.83 (0.72–0.95) | 0.80 (0.65–0.98) | 0.76 (0.59–0.98) |
| 1.16 (0.92–1.47) | 1.08 (0.88–1.33) | 1.08 (0.59–1.95) | 1.01 (0.82–1.24) | Semaglutide SC | 0.95 (0.80–1.14) | 0.94 (0.69–1.28) | 0.94 (0.71–1.24) | 0.84 (0.72–0.98) | 0.81 (0.65–1.01) | 0.77 (0.59–1.00) |
| 1.22 (1.00–1.49) | 1.13 (0.96–1.33) | 1.13 (0.63–2.01) | 1.06 (0.90–1.24) | 1.05 (0.88–1.25) | Finerenone | 0.99 (0.75–1.30) | 0.99 (0.77–1.26) | 0.88 (0.80–0.96) | 0.85 (0.71–1.01) | 0.81 (0.64–1.02) |
| 1.23 (0.90–1.70) | 1.15 (0.85–1.54) | 1.14 (0.61–2.15) | 1.07 (0.80–1.44) | 1.06 (0.78–1.44) | 1.01 (0.77–1.34) | Dapagliflozin | 1.00 (0.70–1.41) | 0.89 (0.68–1.16) | 0.86 (0.63–1.17) | 0.82 (0.58–1.15) |
| 1.24 (0.93–1.65) | 1.15 (0.88–1.49) | 1.14 (0.62–2.12) | 1.08 (0.83–1.40) | 1.06 (0.81–1.40) | 1.02 (0.80–1.29) | 1.00 (0.71–1.42) | Empagliflozin | 0.89 (0.71–1.12) | 0.86 (0.66–1.13) | 0.82 (0.60–1.12) |
| 1.39 (1.16–1.66) | 1.29 (1.13–1.48) | 1.29 (0.73–2.28) | 1.21 (1.05–1.38) | 1.19 (1.02–1.39) | 1.14 (1.05–1.24) | 1.13 (0.86–1.47) | 1.12 (0.90–1.41) | Placebo | 0.97 (0.83–1.12) | 0.92 (0.75–1.14) |
| 1.43 (1.13–1.81) | 1.33 (1.09–1.63) | 1.33 (0.73–2.40) | 1.25 (1.02–1.53) | 1.23 (0.99–1.53) | 1.18 (0.99–1.40) | 1.16 (0.86–1.58) | 1.16 (0.88–1.52) | 1.03 (0.89–1.20) | Exenatide | 0.95 (0.73–1.24) |
| 1.51 (1.14–1.99) | 1.40 (1.09–1.80) | 1.39 (0.76–2.57) | 1.31 (1.02–1.68) | 1.30 (1.00–1.68) | 1.24 (0.98–1.56) | 1.22 (0.87–1.71) | 1.22 (0.89–1.66) | 1.08 (0.88–1.34) | 1.05 (0.81–1.36) | Ertugliflozin |

Table S7. Individual Drug League Table-major kidney disease events

| major kidney disease events | | | | | | |
| --- | --- | --- | --- | --- | --- | --- |
| dapagliflozin | canagliflozin | semaglutide_sc | sotagliflozin | finerenone | dulaglutide | placebo |
| dapagliflozin | 1.23 (0.91,1.67) | 1.44 (1.06,1.94) | 1.25 (0.77,2.03) | 1.47 (1.14,1.89) | 1.55 (1.14,2.11) | 1.76 (1.40,2.22) |
| 0.81 (0.60,1.10) | canagliflozin | 1.17 (0.89,1.53) | 1.02 (0.64,1.62) | 1.19 (0.96,1.48) | 1.26 (0.95,1.66) | 1.43 (1.18,1.74) |
| 0.70 (0.51,0.94) | 0.86 (0.65,1.13) | semaglutide_sc | 0.87 (0.55,1.39) | 1.02 (0.82,1.27) | 1.08 (0.81,1.43) | 1.23 (1.01,1.49) |
| 0.80 (0.49,1.29) | 0.98 (0.62,1.57) | 1.15 (0.72,1.83) | sotagliflozin | 1.18 (0.76,1.82) | 1.24 (0.77,1.98) | 1.41 (0.92,2.15) |
| 0.68 (0.53,0.87) | 0.84 (0.67,1.04) | 0.98 (0.79,1.22) | 0.85 (0.55,1.31) | finerenone | 1.05 (0.84,1.32) | 1.20 (1.09,1.32) |
| 0.65 (0.47,0.88) | 0.80 (0.60,1.05) | 0.93 (0.70,1.23) | 0.81 (0.51,1.29) | 0.95 (0.76,1.19) | dulaglutide | 1.14 (0.93,1.40) |
| 0.57 (0.45,0.71) | 0.70 (0.58,0.85) | 0.82 (0.67,0.99) | 0.71 (0.46,1.08) | 0.83 (0.75,0.92) | 0.88 (0.72,1.08) | placebo |

Table S8. Individual Drug League Table- HHF

| HHF | | | | | |
| --- | --- | --- | --- | --- | --- |
| Ertugliflozin | canagliflozin | dapagliflozin | liraglutide | finerenone | placebo |
| Ertugliflozin | 1.28 (0.79,2.07) | 1.31 (0.69,2.48) | 1.46 (0.87,2.47) | 1.59 (1.01,2.52) | 2.05 (1.34,3.14) |
| 0.78 (0.48,1.27) | canagliflozin | 1.03 (0.61,1.74) | 1.15 (0.78,1.67) | 1.25 (0.94,1.66) | 1.61 (1.28,2.01) |
| 0.76 (0.40,1.44) | 0.97 (0.57,1.65) | dapagliflozin | 1.12 (0.63,1.96) | 1.22 (0.74,2.01) | 1.56 (0.97,2.51) |
| 0.68 (0.41,1.15) | 0.87 (0.60,1.28) | 0.90 (0.51,1.58) | liraglutide | 1.09 (0.77,1.54) | 1.40 (1.03,1.90) |
| 0.63 (0.40,0.99) | 0.80 (0.60,1.06) | 0.82 (0.50,1.36) | 0.92 (0.65,1.30) | finerenone | 1.29 (1.09,1.52) |
| 0.49 (0.32,0.75) | 0.62 (0.50,0.78) | 0.64 (0.40,1.03) | 0.71 (0.53,0.97) | 0.78 (0.66,0.92) | placebo |

Table S9. Individual Drug League Table- Death from any cause

| Death from any cause | | | | | | | |
| --- | --- | --- | --- | --- | --- | --- | --- |
| _liraglutide_ | semaglutide_sc | dapagliflozin | canagliflozin | finerenone | sotagliflozin | exenatide | placebo |
| liraglutide | 1.09 (0.81,1.48) | 1.09 (0.79,1.51) | 1.14 (0.83,1.57) | 1.23 (0.95,1.61) | 1.39 (1.03,1.88) | 1.41 (1.03,1.94) | 1.39 (1.10,1.76) |
| 0.92 (0.68,1.24) | semaglutide_sc | 1.00 (0.75,1.34) | 1.05 (0.79,1.39) | 1.13 (0.90,1.41) | 1.27 (0.98,1.65) | 1.29 (0.97,1.72) | 1.27 (1.05,1.54) |
| 0.92 (0.66,1.27) | 1.00 (0.75,1.34) | dapagliflozin | 1.04 (0.77,1.42) | 1.13 (0.88,1.45) | 1.27 (0.96,1.69) | 1.29 (0.95,1.75) | 1.27 (1.02,1.59) |
| 0.88 (0.64,1.21) | 0.96 (0.72,1.27) | 0.96 (0.70,1.30) | canagliflozin | 1.08 (0.85,1.38) | 1.22 (0.92,1.61) | 1.24 (0.92,1.67) | 1.22 (0.98,1.51) |
| 0.81 (0.62,1.06) | 0.89 (0.71,1.11) | 0.89 (0.69,1.14) | 0.93 (0.72,1.18) | finerenone | 1.13 (0.91,1.40) | 1.15 (0.90,1.46) | 1.13 (1.00,1.27) |
| 0.72 (0.53,0.97) | 0.79 (0.60,1.02) | 0.79 (0.59,1.04) | 0.82 (0.62,1.09) | 0.89 (0.71,1.10) | sotagliflozin | 1.02 (0.77,1.34) | 1.00 (0.83,1.20) |
| 0.71 (0.51,0.97) | 0.77 (0.58,1.03) | 0.77 (0.57,1.05) | 0.81 (0.60,1.09) | 0.87 (0.68,1.11) | 0.98 (0.75,1.30) | exenatide | 0.98 (0.80,1.21) |
| 0.72 (0.57,0.91) | 0.79 (0.65,0.95) | 0.79 (0.63,0.98) | 0.82 (0.66,1.02) | 0.89 (0.79,1.00) | 1.00 (0.83,1.20) | 1.02 (0.82,1.26) | placebo |

Table S10. Individual Drug League Table- CV death

| CV death | | | | | | | | |
| --- | --- | --- | --- | --- | --- | --- | --- | --- |
| liraglutide | semaglutide_sc | empagliflozin | dapagliflozin | finerenone | Ertugliflozin | sotagliflozin | canagliflozin | placebo |
| liraglutide | 1.07 (0.73,1.58) | 1.16 (0.72,1.88) | 1.28 (0.84,1.95) | 1.33 (0.95,1.86) | 1.75 (1.11,2.76) | 1.31 (0.84,2.05) | 1.38 (0.95,2.00) | 1.51 (1.12,2.05) |
| 0.93 (0.63,1.38) | semaglutide_sc | 1.09 (0.69,1.70) | 1.20 (0.82,1.75) | 1.24 (0.93,1.65) | 1.64 (1.08,2.49) | 1.23 (0.82,1.84) | 1.29 (0.93,1.79) | 1.41 (1.11,1.80) |
| 0.86 (0.53,1.39) | 0.92 (0.59,1.44) | empagliflozin | 1.10 (0.69,1.78) | 1.14 (0.76,1.71) | 1.51 (0.91,2.51) | 1.13 (0.69,1.86) | 1.18 (0.76,1.83) | 1.30 (0.89,1.90) |
| 0.78 (0.51,1.18) | 0.83 (0.57,1.22) | 0.91 (0.56,1.46) | dapagliflozin | 1.03 (0.74,1.43) | 1.37 (0.87,2.14) | 1.02 (0.66,1.58) | 1.07 (0.74,1.54) | 1.18 (0.88,1.57) |
| 0.75 (0.54,1.06) | 0.81 (0.61,1.07) | 0.88 (0.58,1.32) | 0.97 (0.70,1.34) | finerenone | 1.32 (0.91,1.92) | 0.99 (0.69,1.42) | 1.04 (0.79,1.36) | 1.14 (0.98,1.33) |
| 0.57 (0.36,0.90) | 0.61 (0.40,0.93) | 0.66 (0.40,1.10) | 0.73 (0.47,1.14) | 0.76 (0.52,1.10) | Ertugliflozin | 0.75 (0.47,1.20) | 0.78 (0.52,1.18) | 0.86 (0.61,1.21) |
| 0.76 (0.49,1.19) | 0.81 (0.54,1.22) | 0.89 (0.54,1.45) | 0.98 (0.63,1.51) | 1.01 (0.71,1.45) | 1.34 (0.84,2.14) | sotagliflozin | 1.05 (0.71,1.55) | 1.15 (0.83,1.59) |
| 0.73 (0.50,1.06) | 0.78 (0.56,1.08) | 0.85 (0.55,1.31) | 0.93 (0.65,1.34) | 0.96 (0.74,1.26) | 1.28 (0.85,1.91) | 0.95 (0.64,1.41) | canagliflozin | 1.10 (0.88,1.37) |
| 0.66 (0.49,0.89) | 0.71 (0.55,0.90) | 0.77 (0.53,1.12) | 0.85 (0.63,1.13) | 0.88 (0.75,1.02) | 1.16 (0.83,1.63) | 0.87 (0.63,1.20) | 0.91 (0.73,1.13) | placebo |

***10 Safety Outcomes***

Among the prespecified safety outcomes, only SAEs included data from all three active drug classes, allowing for a complete network meta-analysis. Other safety outcomes were analyzed using partial network meta-analysis or conventional pairwise meta-analysis due to limited study numbers or incomplete drug class coverage.

***SAE***

Fourteen studies reported SAEs, involving placebo and all three active drug classes (GLP-1RAs, SGLT2is, and nsMRAs), enabling construction of a complete evidence network. A random-effects consistency model was applied for network meta-analysis (Fig. S18). Compared with placebo, GLP-1RAs (RR = 0.85, 95% CI [0.77–0.94]) and SGLT2is (RR = 0.85, 95% CI [0.79–0.92]) significantly reduced the risk of SAEs, while nsMRAs also showed a modest but statistically significant reduction (RR = 0.91, 95% CI [0.85–0.98]). Comparisons between active treatments showed no statistically significant differences: SGLT2is versus GLP-1RAs (RR = 1.00, 95% CI [0.88–1.14]), nsMRAs versus GLP-1RAs (RR = 1.07, 95% CI [0.94–1.22]), and nsMRAs versus SGLT2is (RR = 1.07, 95% CI [0.97–1.19]). Heterogeneity analysis showed no significant between-study heterogeneity (τ² = 0, I² = 0.0%, P = 0.947), indicating good consistency of the pooled estimates.

**Fig.S18 Network meta-analysis reporting RR for SAE in patients with T2DM and CKD**

***Any AE***

A total of 11 studies reported any AE. As GLP-1RAs–related studies did not report this outcome, the network for this endpoint included only SGLT2is, nsMRAs, and placebo. Heterogeneity testing indicated low between-study heterogeneity (τ² = 0; I² = 9.1%; P = 0.359). A random-effects consistency model was applied for the network meta-analysis. Compared with placebo, SGLT2is significantly reduced the risk of any adverse events (RR = 0.83, 95% CI [0.74–0.93]), whereas nsMRAs showed no statistically significant difference compared with placebo (RR = 0.97, 95% CI [0.88–1.07]). Indirect comparisons between active treatments indicated that nsMRAs were associated with a higher risk of AEs compared with SGLT2is (RR = 1.17, 95% CI [1.00–1.36]). Overall, the low heterogeneity suggests good consistency of the pooled estimates for this outcome. (Fig. S19).

**Fig.S19 Network meta-analysis reporting RR for Any ae in patients with T2DM and CKD**

***Adverse Events Leading to Discontinuation***

A total of 12 studies reported adverse events leading to treatment discontinuation, involving placebo and all three active drug classes (GLP-1RAs, SGLT2is, and nsMRAs), allowing construction of a complete network. Heterogeneity testing indicated low between-study heterogeneity (τ² = 0.0055; I² = 10.5%; P = 0.346). A random-effects consistency model was applied for the network meta-analysis. Compared with placebo, GLP-1RAs showed no statistically significant difference in the risk of discontinuation due to adverse events (RR = 1.12, 95% CI [0.87–1.43]). SGLT2is showed a trend toward reduced risk, but this did not reach statistical significance (RR = 0.86, 95% CI [0.73–1.02]). In contrast, nsMRAs showed a trend toward increased risk, approaching statistical significance (RR = 1.19, 95% CI [0.99–1.42]). Comparisons between active treatments showed no significant differences between SGLT2is and GLP-1RAs (RR = 0.77, 95% CI [0.57–1.04]) or between nsMRAs and GLP-1RAs (RR = 1.06, 95% CI [0.78–1.44]). However, nsMRAs were associated with a significantly higher risk of discontinuation compared with SGLT2is (RR = 1.38, 95% CI [1.08–1.76]). Overall, the low heterogeneity across studies indicates good robustness of the results for this outcome. (Fig. S20).

**Fig.S20 Network meta-analysis reporting RR for ae leading to discontinuation in patients with T2DM and CKD**

**Hyperkalemia**

A total of 6 studies reported hyperkalemia outcomes, involving SGLT2is, nsMRAs, and placebo-controlled evidence (Fig. S21). Compared with placebo, SGLT2is significantly reduced the risk of hyperkalemia (RR = 0.76, 95% CI [0.62–0.92]), whereas nsMRAs significantly increased the risk (RR = 2.19, 95% CI [1.93–2.48]). Indirect comparisons between active treatments indicated that nsMRAs were associated with a significantly higher risk of hyperkalemia compared with SGLT2is (RR = 2.89, 95% CI [2.30–3.63]). Heterogeneity testing suggested low between-study heterogeneity (τ² = 0.0055; I² = 10.5%; P = 0.346).

**Fig.S21 Network meta-analysis reporting RR for hyperkalemia in patients with T2DM and CKD**

***Severe Hypoglycemia***

For the outcome of severe hypoglycemia, the network meta-analysis included 7 studies, involving GLP-1RAs, SGLT2is, and placebo-controlled evidence (Fig. S22). Compared with placebo, neither GLP-1RAs (RR = 0.78, 95% CI [0.45–1.34]) nor SGLT2is (RR = 0.76, 95% CI [0.32–1.83]) showed a statistically significant difference in the risk of severe hypoglycemia. Indirect comparisons between active treatments also revealed no significant difference, with an RR of 0.98 (95% CI [0.34–2.80]) for SGLT2is versus GLP-1RAs. Heterogeneity testing indicated moderate between-study heterogeneity (τ² = 0.1082; I² = 41.6%; P = 0.1620), suggesting considerable uncertainty in the pooled estimates and warranting cautious interpretation of this outcome.

**Fig.S22 Network meta-analysis reporting RR for severe hypoglycemia in patients with T2DM and CKD**

*Genital Infection*

A total of 7 studies reported genital infection events, all of which were direct comparisons between SGLT2is and placebo. Heterogeneity testing indicated moderate between-study heterogeneity (I² = 50.4%, τ² = 0.2056, P = 0.06); therefore, a conventional random-effects pairwise meta-analysis was performed. The results showed that, compared with placebo, SGLT2is significantly increased the risk of genital infections (RR = 2.41, 95% CI [1.50–3.88]) (Fig. S23).

**Fig.S23 pairwise meta-analysis——Genital Infection**
